# Pain-related toxins in scorpion and spider venoms: a face to face with ion channels

**DOI:** 10.1590/1678-9199-JVATITD-2021-0026

**Published:** 2021-12-06

**Authors:** Sylvie Diochot

**Affiliations:** 1 Institut de Pharmacologie Moléculaire et Cellulaire (IPMC), Centre National de la Recherche Scientifique (CNRS) UMR 7275 et Université Côte d’Azur (UCA), 06560 Valbonne, France. Institut de Pharmacologie Moléculaire et Cellulaire Centre National de la Recherche Scientifique Université Côte d’Azur Valbonne France

**Keywords:** Venom, Toxin, Spider, Scorpion, Pain, Ion channels

## Abstract

Pain is a common symptom induced during envenomation by spiders and scorpions. Toxins isolated from their venom have become essential tools for studying the functioning and physiopathological role of ion channels, as they modulate their activity. In particular, toxins that induce pain relief effects can serve as a molecular basis for the development of future analgesics in humans. This review provides a summary of the different scorpion and spider toxins that directly interact with pain-related ion channels, with inhibitory or stimulatory effects. Some of these toxins were shown to affect pain modalities in different animal models providing information on the role played by these channels in the pain process. The close interaction of certain gating-modifier toxins with membrane phospholipids close to ion channels is examined along with molecular approaches to improve selectivity, affinity or bioavailability *in vivo* for therapeutic purposes.

## Background

Scorpions and spiders are venomous animals belonging to the arachnid class of arthropods. These small elusive arthropods have a long history of terrifying their victims, including humans. No less than 49,000 species of spiders (https://wsc.nmbe.ch/) and 1,900 species of scorpions can be found throughout the world, with an especial diversity and density in sub-tropical and tropical regions. Their venom, a tool for catching prey or defending against aggressors, is composed of a few dozen to several hundred toxins having high specificity and affinity for tissues. Depending on the venom considered and the dose injected, these toxins act together to cause paralysis, which facilitates the escape, or the sudden death of the aggressor. Arachnid venoms contain small molecules (ions, amino acids, monoamines, polyamines), a great number of peptides but also proteins and enzymes. Scorpion venom peptides mainly consist of neuropeptides, cardiopeptides and antimicrobial peptides with cytotoxic activities [1], whereas spider venom is composed of more neuroactive peptides, a few cardiotoxic and antimicrobial peptides, and enzymatic proteins contributing to paralysis, death and tissue digestion during prey feeding [2-4].

## Scorpion Stings and Spider Bites

**Figure 1.**
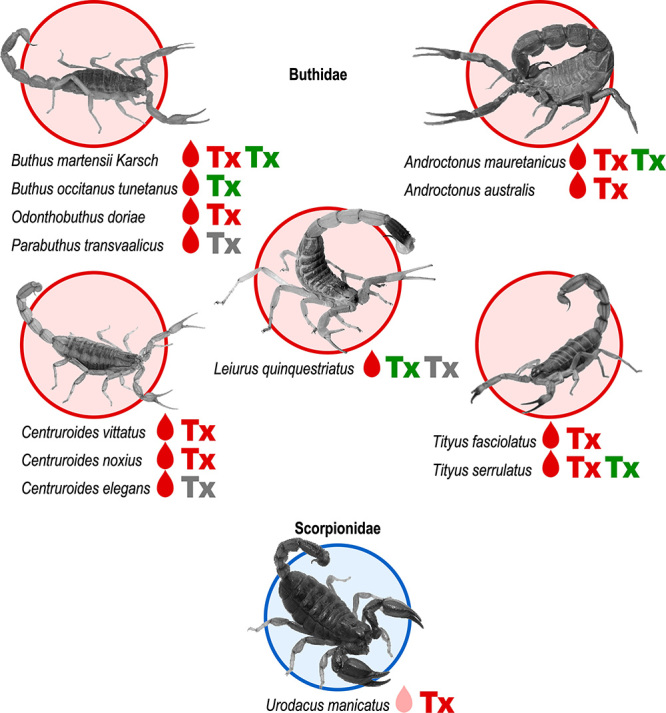
Pain-related scorpion species. Scorpion species of medical importance are circled in red, those that are harmless to humans are circled in blue. Red drop: scorpions with highly painful stings; pink drop: scorpions whose sting is mildly painful. **Tx**: presence of pro-algic toxins in the venom; **Tx**: presence of antinociceptive toxins in the venom; **Tx**: presence of toxins having an effect only on pain-related channels.

With more than 1.2 million stings a year and more than 3250 deaths, scorpionism is a major public health problem in sub-tropical areas worldwide [5] despite the fact that less than 25 species are considered dangerous to humans. The “Old Word” (Africa) species of medical interest belong to *Andoctonus*, *Buthus, Hottentota*, *Leiurus* genera while “New World” (America) species are part of *Centruroides* and *Tityus* genera, all in the Buthid family (Figure 1). Severe envenomation in humans primarily occurs in tropical regions and during hot seasons (North and Sub-Saharan Africa, Middle East, Asia, Latin America), with stings inoculating a few microliters of venom. Incidence (number of scorpion stings per 100,000 inhabitants) varies in each country as well as within rural and urbanized areas (250 and 15 respectively in Morocco), with the majority of stings occurring in the summer months. In Tunisia, before the extensive use of antivenom, the mortality rate reached 6.67 per 100 000 inhabitants with an incidence of 1500 [5]. Alarming cases predicting severe envenomation (5% of envenomation cases) include local symptoms (pain and paresthesia) associated with systemic gastrointestinal, respiratory, cardiopulmonary and neurological symptoms [6-8]. Envenomation is particularly dangerous for children under 15 years where bites often result in acute pulmonary edema leading to death [9].

**Figure 2.**
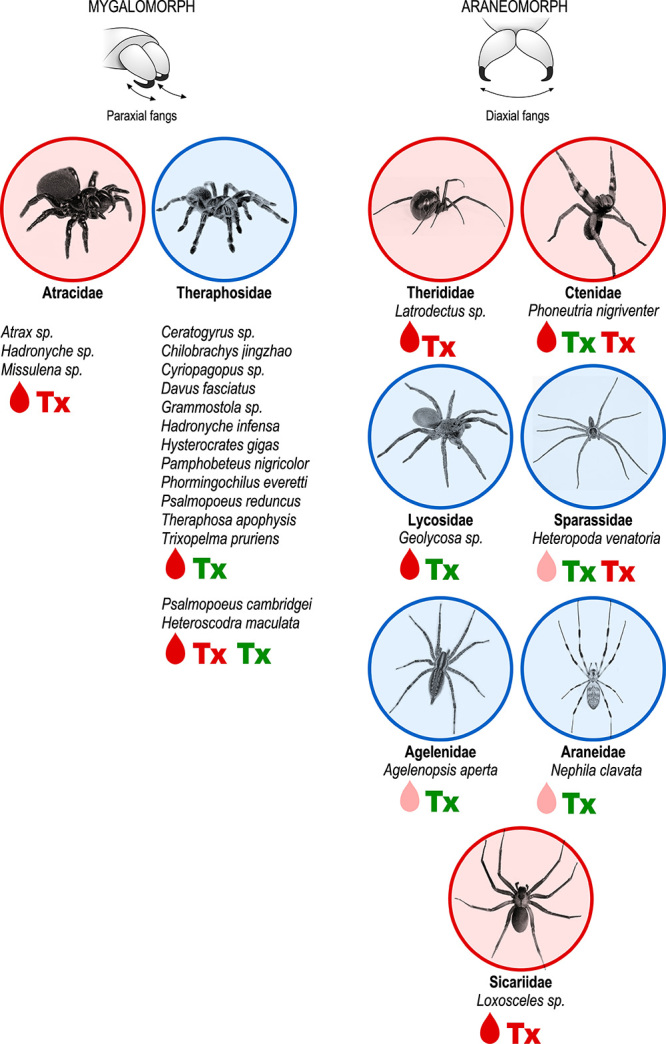
Pain-related spider species. Mygalomorph and araneomorph spiders are differentiated by their morphologic chelicerae position. Mygalomorph have primitive orthognath position, with parallel fangs, whereas araneomorph have labidognath position, in which their fangs move side to side, like a pair of scissors. Spider species of medical importance are circled in red, those that are harmless to humans are circled in blue. Red drop: spiders with highly painful bites, some (*Latrodectus* and *Loxosceles*) have a late onset of pain (a few hours after the bite); pink drop: spiders whose bite is mildly painful. **Tx**: presence of pro-algic toxins in the venom; **Tx**: presence of antinociceptive toxins in the venom and/or toxins with high affinity for pain-related channels.

Envenomation by spider bites is considered dangerous for humans in a few species. Large spiders and in particular mygalomorphs are not the most dangerous, except for the genera *Atrax* or *Hadronyche* (Atracidae, Australia) and *Missulena* (Actinopodidae, Australia) [10]. In the araneomorph group, genera *Phoneutria* (Ctenidae, South America), several species of *Loxosceles* (Sicariidae, mainly found in North and South America) and *Latrodectus* (Theridiidae, in South and sub-tropical regions all other the world) are responsible for severe envenomation but rarely are these fatal when treated symptomatically or with anti-venom serology [11] (Figure 2).

A necrotizing araneism is described in bites by several species of *Loxosceles*, without neurotoxicity, leading to skin lesions with necrosis sometimes requiring skin grafts, or thrombocytopenia responsible for hemorrhage. In severe forms, a viscerocutaneous syndrome characterized by fever, hemolytic jaundice, and nephropathy can result in the death of the victim [11,12]. A neurotoxic araneism is described in cases of bites by species of *Atrax*, *Missulena*, *Phoneutria* and *Latrodectus* genera associating respiratory, cardiac and digestive symptoms. These cases of envenomation present a common picture of localized pain, discomfort, nausea, sweating, vomiting, tachycardia, hyper/hypotension, and muscle fasciculations. These symptoms worsen with the onset of dyspnea, cardiovascular collapse, respiratory failure, which can be fatal [13,14]. More characteristic symptoms of *Latrodectus* bites include sustained muscle cramps, mental confusion, and abdominal pain [10,15].

## Pain Processing During Envenomation

Cases of envenomation by arachnid bites or stings, always provoke first peripheral symptoms due to the effect of the venom toxins on the excitable nerve endings. Some spider venoms, such as *Loxosceles*, cause additional cytolytic effects characterized by tissue necrosis due to the presence of enzymes such as phospholipases D (sphingomyelinases D) highly responsible for venom dermonecrotic activity [12,16,17]. Scorpion stings always cause sharp, constant and lasting pain, regardless of whether the species is dangerous or not [5,18]. In victims, the pain is immediate, and very intense, first local, then loco-regional, it can subside over short periods of time, which may suggest an improvement in general condition, but often remains intense for one to two days [5,19]. In the case of poisoning by dangerous species, the pain will gradually be accompanied within a few hours by other symptoms such as nausea, sweating, vomiting, agitation, malaise, and hypertension [20].

A comparative work between Buthidae and Vaejovidae venom suggests that stings of Buthid scorpions, the most dangerous for humans, are more painful to mammals than sting from non-Buthid scorpions (*Vaejovis spiniferus*) [21]. Observations of peripheral venom injected to rodents show immediate pain characterized by continuous flinching and licking on the injected paw. Thermal and mechanical hypersensitivity to pain associated with edema, which reflects inflammation, can extend to several days or weeks [22,23].

The pain felt when bitten by a spider is undoubtedly more variable, sometimes discreet but becoming intense dependent upon the species considered. The pain profile can evolve during the course of the symptomatology ranging from simple localized sharp pain, to radiating pain towards the limbs, or generalized pain associating a burning sensation (evoking inflammation), muscle cramps, or itching. In the case of spiders, the intensity of the pain is by no means synonymous with a worrying evolution [10,11,24]. Spider venoms are very complex in nature and the combined effect of all the venom components induce a wide range of neurotoxic symptoms that resemble those described for scorpion envenomation.

The immediate, acute pain that appears following a spider bite or scorpion sting is the consequence of the effect of toxins on receptors in the peripheral nervous system. The pain sensation typically originates in primary sensory afferent neurons known as nociceptors, through which pain signals are relayed from the periphery to central nervous system. The hyperexcitability of primary sensory neurons is one of the mechanisms that can lead to exaggerated sense of pain, such as spontaneous pain and hyperalgesia [25-27].

These venoms contain small molecules such as monoamines (dopamine, epinephrine, norepinephrine, histamine, octopamine, serotonin, tyramine) which, by activating their specific receptor, are pro-algesic [28,29]. Others, such as certain neurokinins, cytokines, ATP, NO, excitatory amino acids also modulate pain and inflammation [26,30]. Most of these venoms contain peptides with excitatory activity, which target ion channels on sensory neurons and initiate the rapid nociceptive response. Enzymes such as sphingomyelinases, phospholipase A2, hyaluronidases, induce an inflammatory response causing the release of pro-inflammatory cytokines and lipid mediators, leading to later onset of secondary pain [12,16]. Protease inhibitors have also been involved in the prolonged pain behavior induced by scorpion crude venom [31]. 

 In scorpion venoms, a few toxins with analgesic activity in mammals have been identified and their molecular target determined [32-34]. The situation is quite different for spider venoms, where in parallel with the discovery of excitatory toxins inhibiting potassium channels or activating sodium channels, a large number of peptides with analgesic properties have been isolated [35-37]. These analgesic toxins target ion channels involved in pain transmission pathways and are becoming essential tools for the study of pain mechanisms [38,39]. They could also be promising, as drug candidates for treating various types of intractable pain, and to enrich the existing pharmacopoeia.

## Ion Channels and Pain

Ion channels are ubiquitous transmembrane proteins permeable to ions and activated by a variety of stimuli (voltage, temperature, ligands, pH...) specific to each ion channel family. They represent the molecular basis of the mechanisms of propagation of action potentials of excitable cells and thus modulate neuronal, muscular, and cardiac physiology [40,41]. More than a hundred genes have been cloned encoding ion channels with a high diversity of structure, functions, regulations and pharmacology [42,43]. In the vertebrate nervous system, certain subtypes of ion channels are involved in the detection, transmission and integration of different nociceptive stimuli [44]. The best characterized ion channels in the context of pain belong to the super family of voltage-dependent sodium channels (Nav), voltage-dependent calcium channels (Cav), Acid-Sensing Ion Channels (ASICs), some potassium channels, channels in the Transient Receptor Potential (TRP) family, ionotropic (P2X, Serotonin and Glutamate) receptors, and certain mechanosensitive channels (SACs, Piezo) [44,45]. The role of ion channels in pain was demonstrated in particular with the use of specific pharmacological tools such as animal toxins that activate or block their functioning, when the gene deletion models (knockout (KO) or knockdown mice) did not reveal a marked phenotype [25].

### Voltage-gated sodium channels

In the Nav family, homologous mammalian genes encode nine members, Nav1.1 to Nav1.9. The TTX-sensitive (TTX-S) Nav1.7, and the TTX-resistant (TTX-R) Nav1.8 and Nav1.9 channels, largely expressed in sensory neurons, are recognized as the most important contributors to the control of nociceptive responses in rodents and humans [46-49] . Mutations in human genes, encoding the α-subunit of Nav1.7, Nav1.8 and Nav1.9, were found to be responsible for congenital pain insensitivity (loss of function), erythromelalgia (gain of function) and paroxysmal extreme pain disorders, different peripheral neuropathies [50-52].

The TTX-S Nav1.1, Nav1.3 and Nav1.6 were recently implicated in pain. Nav1.1 channels are related to mechanical pain [53], Nav1.3 is involved in cold thermosensation and mechanosensation [54] and Nav1.6 contributes to the development and maintenance of neuropathic pain [55,56].

### Voltage-gated potassium channels

 In the large family of potassium channels encoded by more than 80 genes, voltage-gated K^+^ channels (Kv) include various members classified into Kv1-Kv12 subfamilies based on their biophysical and pharmacological properties [57]. In the nervous system, some Kv contribute to the shape and frequency of action potentials that modulate axonal conduction [58-60]. The membrane hyperpolarization induced by K channels openers is able to regulate and stabilize neuron excitability, and may provide antinociceptive effects in sensory pathways. In arachnid venoms, toxins that activate Nav channels and block Kv channels are often found simultaneously, and act synergistically to depolarize excitable membranes permanently and produce neurotoxicity and pain [61]. The Kv4 family, which is targeted by some spider and scorpion toxins, is implicated in the modulation of pain. Specific inhibition of Kv4.2 by a scorpion toxin controls the mechanical nociception [62]. A dysfunction of Kv4.3 in trigeminal ganglion neurons has recently be shown to impact neuropathic pain associated with cold hypersensitivity [63].

### Voltage-gated calcium channels

Cav channels control key functions in excitable cells including transmitter release, hormone secretion, gene expression or muscle contraction [64]. They comprise T-type channels (encoded by Cav3.1 to 3.3 genes), L-type (Cav1.1 to 1.4), N-type (Cav2.2), P/Q-type (Cav2.1) and R-type (Cav2.3) channels. Cav2.2, which is predominantly expressed in presynaptic terminals in the peripheral and central nervous systems, is essential in neurotransmitter release [65]. It contributes to pain transmission in particular in neuropathic and inflammatory pain [66,67]. The role of Cav2.3 in inflammatory pain [68] and in nociceptive transmission during neuropathic pain [69] was highlighted by knockout studies. Cav3.2 channels, which have a large expression in nociceptors, are important contributors for nociception as shown by knockdown studies in rodents [70].

### ASIC channels

Acid Sensing Ion Channels (ASIC) are voltage-insensitive cation channels activated by extracellular protons, generating mainly transient inward sodium currents [71]. To date, 4 genes encoding 6 isoforms (ASIC1a, ASIC1b, ASIC2a, ASIC2b, ASIC3 and ASIC4) can combine to form homo- or hetero-trimers highly expressed in CNS and PNS [72]. Their activation induces neuronal depolarization and is associated with several physiological and physiopathological processes such as neuronal plasticity, neuroprotection, cancer and nociception [73,74]. ASIC1a contribute to acute, inflammatory and neuropathic pain. Use of specific pharmacological venom peptides in rodent pain models showed that their blockade causes analgesia and their activation elicits acute pain [75-77]. The role of ASIC1 in migraine and the therapeutic potential of ASIC1 inhibitors in this pathology were also recently shown [78].

Heteromultimeric ASIC1a/ASIC2a channels contribute to opioid-independent analgesia in the CNS [77] while ASIC2b combined to ASIC1a contribute to acute, inflammatory and neuropathic pain [79]. ASIC1b, which are only expressed in the PNS, contribute to the modulation of acute, inflammatory and probably neuropathic pain [73,77,80]. In sensory neurons, ASIC3 are important actors for skin and muscle pain [81,82].

### Transient receptor potential channels

Transient receptor potential (TRP) channels are polymodal receptors widely expressed in sensory neurons in mammals, permeable to Ca^2+^ and Na^+^. They detect changes in temperature, light, acidity or osmolarity. Different subtypes (TRPV1, TRPV3, TRPV4, TRPA1, TRPM2, TRPM3 and TRPM8) are activated by painful stimuli in various modalities [83]. TRPV1 opens under nociceptive stimuli such as capsaicin, heat and acidity. Mice with TRPV1 gene deletion are less responsive to noxious heat, revealing that the TRPV1 channel is essential for selective modalities of sensation of pain and for tissue injury induced thermal hyperalgesia [84,85]. TRPV3 punctual mutations underlined their role in migraine, fibromyalgia and erythromelalgia [86-88]. TRPV4 are related to sensory or motor neuropathies associated with pain [89]. Genetic deletion of TRPV4 in mice also highlighted their role in inflammatory pain. 

Cooling agents such as menthol and icilin activate the sub family of TRPM8 channels expressed in sensory neurons. TRPM8 is as a cold sensitive receptor, its pharmacological blockade and genetic deletion showed its role in thermoregulation and in cold hypersensitivity in neuropathic pain [90]. TRPM3 is another temperature sensitive channel expressed in heat afferent neurons whose genetic deletion results in reduced response to noxious heat as well as to inflammatory thermal hyperalgesia [91].

TRPA1 channels are activated by various “burning” agents such as mustard, cinnamon, wasabi, which promote the sensation of pain. Their genetic deletion or pharmacological blockade induces a loss of reaction in chemical, mechanical and inflammatory nociceptive pain models [92]. TRPA1 also seems to have a role in the perception of noxious cold responses associated with or without inflammation [93] and is associated to pain during diabetic neuropathy [94].

### Purinergic P2X ion channels

Purinergic P2X ion channels, include seven members (P2X1 to P2X7) permeable to Ca^2+^ and activated by ATP. Some of these channels expressed in sensory neurons such as P2X2 and P2X3, can associate in heterotrimers, and have a role in the initiation of nociception [95]. Thus, ATP by activating P2X3 receptors can induce mechanical allodynia in rats [96]. Knock out and knock down studies revealed that P2X3 is involved in inflammatory and neuropathic pain in rats [97,98].

Opening of P2X4 is known to induce allodynia during neuropathic pain situations [99]. In the same way, P2X7 receptors stimulated during microglial inflammation are involved in the development of neuropathic and inflammatory pain [100].

It should be mentioned that arthropod venoms include nucleosides such as ATP, ADP, AMP and adenosine that can activate purinergic receptors and contribute to early pain processing during envenomation.

### Serotonin ionotropic receptors

Serotonin (5-HT), a neurotransmitter widely released in the nervous system, participates to the modulation of pain, sleep and mood. 5-HT, by acting on its receptors, a family including seven members, 5HT1 to 5HT7, can promote or suppress pain sensations [101]. In the ionotropic 5-HT3 receptor family, five subtypes 5-HT3A to E are described in both the central and peripheral nervous system, and are permeable to Ca^2+^ and Na^+^. Several studies have shown that central injections of the 5-HT3 receptor antagonists have antinociceptive effects on neuropathic pain while activators induce neuronal hyperexcitability and pain hypersensitivity [102,103]. Other studies demonstrated opposite results on pain, using 5-HT3 agonist or antagonists, that could be attributed to different pain models, drug concentrations and mode of administration [104]. Serotonin reuptake drugs, developed by pharmaceutical companies, are known to be effective against several pain syndromes, in particular in migraine and neuropathic pain.

### Piezo channels

The Piezo family includes two mechanosensitive cationic channels, Piezo1 and Piezo2, found in the bladder, colon, kidney, lung and skin. They are mainly permeable to Ca^2+^, less to Na^+^, K^+^ and Mg^2+^, and their function is related to touch and mechanical pain sensations [105]. Piezo2 strongly expressed in DRG sensory neurons seems to contribute to somatosensory mechanotransduction and allodynia during inflammation [106]

### Glutamate ionotropic receptors

The glutamate ionotropic receptors are opened by glutamate, and localized mainly at the postsynaptic level in the nervous system. At the spinal cord level, they control fast sensory transmission and plasticity but also the generation of long-term memory in the cerebral cortex. They are differentiated into NMDA (N-methyl-D-aspartate), AMPA (α-amino-3-hydroxy-5-methyl-4-isoxazoleproprionic acid) and kainate (KA) receptors permeable to Na^+^ or to Ca^2+^ [107]. Glutamate is the major neurotransmitter between primary afferent fibers and dorsal horn neurons in the spinal cord and contributes to the genesis of excitatory postsynaptic currents (EPSCs) that play a role in pain and itch transmission via AMPA, KA and NMDA receptors [108]. Glutamate is important for nociceptive sensitization in the brain where AMPA, KA and NMDA receptors also play an important role in chronic inflammatory and neuropathic pain [109,110].

## Scorpion Toxins Interacting with Pain-Related Ion Channels

During envenomation in humans, a majority of scorpion venom toxins target Nav and Kv channels, acting synergistically, to induce neurotoxic symptoms of hyperexcitability (hypertension, cardiac hyperactivity, hypersecretion of neurotransmitters). Toxins that bind to Nav channels are 60 to 70 amino acid peptides with a conserved structural scaffold consisting of an alpha-helix, and three-stranded beta sheets connected by four disulfide bridges in a βαββ topology. Some are more or less selective for insect versus mammalian Nav channels with sub-micromolar affinities, and they are classified into 2 types (α- and β-toxins) according to their mode of action on mammalian Nav [111-113]. 

Toxins from scorpions with known pain-related ion channels and/or pain-related effects are listed in Table 1. Their mode of action on ion channels are indicated in relation to their classification. Classical α- or β-toxins are highly specific for mammals, whereas α- or β-like toxins are active on both mammals and insects. Finally, insect α- and insect β-toxins are specific for insects and not toxic for mammals even at high concentrations. Moreover, α/β-toxins are those targeting site 3 and 4, simultaneously affecting fast inactivation and steady state activation of Na^+^ currents.

**Table 1.** Scorpion toxins active on ion channels related to pain.

Scorpion species	Toxin	Action mode	Ion channel	Type of effect	Pain phenotype [references]
*Androctonus australis*	AaH II	α-toxin	TTX-S Nav (Nav1.7)	Pain	Acute, mechanical and thermal pain (*ipl*) [123]
*Androctonus mauretanicus*	Amm VIII	α-toxin	TTX-S Nav Nav1.7	Pain	Acute, mechanical and thermal pain (*ipl*) [123]
	anatoxin Amm VIII	α-toxin	*Nav1.2*	Analgesia	Thermal (*ip*) pain [125]
*Buthus martensii* Karsch	BmK I	α-toxin	Nav1.6 (pot) Nav1.8 (pot)	Pain	Acute, inflammatory thermal pain, mechanical hyperalgesia (*ipl*) [115,116,118-120]
	BmK IT2	β-toxin	TTX-R &TTX-S Nav (inh)	Analgesia	Acute, inflammatory pain, thermal hyperalgesia (*it*, *ipl*) [133-136]
	BmK IT-AP	Excitatory insect β-toxin	ND	Analgesia	Visceral pain (*iv*) [138,150]
	BmK IT AP3	Depressant insect toxin	ND	Analgesia	Visceral pain (*iv*) [138]
	BmK AS BmK AS-1	β-toxin	TTX-R &TTX-S Nav (inh)	Analgesia	Visceral (*ip*, *iv*), thermal(*iv*), Inflammatory (*it*) pain, mechanical hyperalgesia (*ipl*) [32,131,132]
	BmK AGP-SYPU1&2	α/β-toxin	*Nav1.4*; *Nav1.5*; Nav1.7	Analgesia	Visceral (*ip*, *iv*) pain [339]
	BmK AGAP-SYPU1&2	α/β-toxin	ND	Analgesia	Visceral (*ip*, *iv*), thermal (*iv*) pain [340]
	BmK AGAP	α/β-toxin	*Nav1.4*, *Nav1.5* Nav1.7, Nav1.8 (inh)	Analgesia	Visceral (*ip*), inflammatory (*ipl*, *iv*), thermal (*iv*) pain [146,147]
	BmK AngM1		Nav (inh)	Analgesia	Visceral pain (*iv*) [148]
	BmK AngP1	Excitatory insect toxin	ND	Analgesia	Visceral pain (*iv*) [138]
	ANEP toxin	β-toxin	Nav1.7 (inh)	Analgesia	Visceral pain (*iv*) [33]
	BmP01		TRPV1 (act) *Kv1.3* (inh)	Pain	Acute pain (*ipl*) [160,161]
*Buthus occitanus tunetanus*	Bot AF	β-like-toxin	ND	Analgesia	Heat (*ip*, *it*), inflammatory (*ipl*), visceral (*ip*) pain [154]
*Centruroides elegans*	CeII8	β-toxin	Nav1.7 (inh)	ND	[139]
*Centruroides vittatus*	Cv IV4	α-toxin	*Nav1.2*, Nav1.3, *Nav1.4*, Nav1.7 (pot)	Pain	Acute pain (*ipl*) [21]
*Centruroides noxius*	Cn2	β-toxin	Nav1.6 (act)	Pain	Acute pain, mechanical cold allodynia (*ipl*) [143,144]
*Leiurus quinquestriatus*	LqqIT2	Insect β-toxin	ND	Analgesia	Thermal pain (*ip*) [125]
	LqhIII	α-like toxin	Nav1.7 (pot)	ND	[124]
*Odonthobuthus doriae*	OD1	α-like-toxin α/β-toxin	Nav1.7, Nav1.3 *Nav1.4*, Nav1.6 (pot)	Pain	Acute pain (*ipl*) [126-129]
*Parabuthus transvaalicus*	Kurtoxin	α-toxin	Cav3.1, Cav3.2, Cav2.2 (inh); *Nav1.2* (pot)	ND	[155,156]
*Tityus fasciolatus*	Tf2	β-toxin	Nav1.3 (act)	Pain	Acute pain (*ipl*) [140,317]
*Tityus serrulatus*	Ts2	α-toxin	Nav1.3, Nav1.6, Nav1.7 (pot)	ND	[142]
	TsNTxP		ND	Analgesia	Acute, heat, inflammatory, neuropathic pain (*it*) [153]
	Ts8 (TsK2 or TsTx-Kβ)	β-KTx	Kv4.2 (inh)	Pain	Acute pain mechanical hyperalgesia (*ipl*, *it*) [62]
*Urodacus manicatus*	WaTx		TRPA1 (act)	Pain	Acute pain (*ipl*) [163]

ANEP: anti-neuroexcitation peptide; BmK AGAP: *Buthus martensii* Karsch-analgesic-antitumor peptide; pain-related ion channel and other high-affinity channels (in italics) are indicated; inh: inhibition; act: activation; pot: potentialisation; ND: not done. *it*: intrathecal; *ip*: intraperitoneal; *ipl*: intraplantar; *iv*: intravenous. Pain phenotypes and injection routes experimented in rodents are indicated in brackets.

Scorpion α-toxins, also known as “excitatory toxins”, increase the peak and slow down the inactivation kinetics of sodium currents by binding to the site 3 (S4 segment of domain IV voltage sensor) of the TTX-S and TTX-R Nav channels [114]. Among them, neurotoxins possessing nociceptive effects like BmK I, a major peptide isolated from the Chinese *Buthus martensii* venom that is non-lethal for mammals, have been shown to induce spontaneous pain and hyperalgesia upon subcutaneous injections in rat hind paw [115,116]. Its activity involves the TTX-S Nav1.6 and TTX-R Nav1.8 channels which are over-expressed when BmK I is administered to rodents [117]. BmK I potentiates Nav1.6 and Nav1.8 currents revealing an important role for these channels in the modulation of spontaneous pain and mechanical allodynia [118-120]. During the process of hyperalgesia and pain sensitization induced by BmK I, it has been shown that both neuronal 5-HT3AR signaling pathway and microglia, with up-regulation of P2X7 receptors and interleukin 1β, are activated [121,122].

The deadly North-African scorpions *Androctonus australis* and *A. mauretanicus* also have α-toxins (AaH II and Amm VIII) able to induce mechanical and thermal pain hypersensitivity after peripheral injections in mice [123]. These toxins are particularly active on hNav1.7 currents, slowing their inactivation kinetics. Other pain-inducing α-toxins such as CvIVA (isolated from *Centruroides vittatus*) or LqhIII (*Leiurus quinquestriatus*), slow the fast inactivation of Nav1.7 channels [21,124]. Controversial effects have been shown using peripheral intraperitoneal (*ip*) injections of Amm VIII in mice that induce analgesic effects on hot plate pain model [125]. In this study, the authors suggest that activation of an opioid-dependent diffuse noxious inhibitory control could be responsible for the pain relief.

The α-toxin OD1, purified from the Iranian scorpion *Odonthobuthus doria*e venom, is known to inhibit fast inactivation of mammalian Nav1.4, Nav1.6 and Nav1.7 channels with nanomolar EC_50_ values but it also has a β-toxin activity on Nav1.4 and Nav1.6 channels [126]. OD1 also affects the inactivation kinetics of insect Nav channels [127,128]. Mutagenesis study using a triple mutant showed that 3 residues (Asp9, Asp10, Lys11) in the reverse turn region (region 8-12) contribute to sodium channel selectivity, the mutant being more selective to Nav1.7 over Nav1.6 [126]. In a recent study, using synthetic analogs of OD1 that are more potent on Nav1.7, it was shown that the effect of OD1 on Na^+^ current inactivation is due to prolonged flickering between open and closed states [129]. OD1 has become a tool classically used to study acute pain behavior after peripheral administration in rodents.

The so-called “depressant scorpion β-toxins”, by binding to the Nav site 4, and specifically on the extracellular loop of segments S3-S4 of domain II, are able to make them hyperactivable by shifting the activation curves of the currents towards negative potentials. Cells then have a long-lasting hyperexcitability [130]. Some of these toxins have analgesic properties. For instance, peripheral intraplantar (*ipl*) injections of BmK AS, isolated from *Buthus martensii,* display antinociceptive effects in a model of thermal and mechanical hyperalgesia induced by carrageenan in rats [32,131]. Moreover, central intrathecal (*it*) injections of BmK AS suppress spontaneous nociceptive behavior in rat formalin tests [132]. In the same venom, BmK IT2, a depressant insect selective toxin, reduces thermal pain and hyperalgesia after a peripheral injection in normal and inflamed rats [133,134]. The two peptides BmK AS and BmK IT2 have been further shown to block TTX-R and TTX-S Na^+^ conductances in rat sensory neurons, effects correlated with their analgesic effects [32,132,135,136]*.* Mutagenesis studies have proposed that critical aromatic residues (Trp, Tyr, Phe), that compose the hydrophobic active surface of the toxin, are involved in pharmacological blockade and in the analgesic activity of BmK AS [137].

Interestingly, other insect-selective toxins devoid of toxicity to mammals have been purified from *Buthus martensii* venom, which are also able to induce analgesic effects in mice. BmK IT-AP and BmK AngP1 are excitatory insect toxins, with analgesic effects on the acidic twisting mice pain model [138]. However, BmK AngP1 analgesic effect is 5 times weaker than that of BmK IT-AP, although its toxicity to insects is twice as strong. This means that analgesic effects cannot be correlated with insect toxicity.

The first β-toxin blocker of Nav1.7 is CeII8, isolated from the *Centruroides elegans* venom. It inhibits the peak sodium current, but its activity on pain models has not yet been investigated [139].

Until recently, a few scorpion toxins have been isolated that specifically target one of the nine mammalian Nav channel isoform. Tf2, a β-toxin that activate hNav1.3 channels involved in epilepsy and pain perception with a high specificity, was purified from the Brazilian scorpion *Tityus fasciolatus*. Tf2 (1µM) shift hNav1.3 activation voltage to much more negative values, effectively opening the channel at resting membrane potentials [140]. A more recent study reveals that Tf2 is also able to activate a hNav1.9 chimera at µM concentration [141]. Its activity on Nav1.3 is comparable to that of Ts2, another β-toxin isolated from *Tityus serrulatus* venom, but Ts2 is less specific, as it affects activation or fast inactivation of other pain-related Nav channel isoforms [142]. Peripheral (*ipl*) injections of Tf2 in mice causes spontaneous flinching and swelling, but this painful behavior is also observed when Nav1.3 is inhibited in mice [141]. Moreover, a single mutation on a synthetic analogue of Tf2, Tf2[S^14^R], is able to remove the excitatory activity of the toxin, and to be much less active on Nav1.3, while it retains its *in vivo* pain activity. This means that Nav1.3 is not the unique target for pain inducing effects of Tf2 and suggests an off-target activity [141].

The β-toxin Cn2 isolated from *Centruroides noxius* venom is a specific activator of Nav1.6 channels, which play an essential role in pain transmission in peripheral sensory neurons. Cn2 promotes and enhances Nav1.6 current (EC_50_= 39nM) in large mice DRG neurons and induces a significant increase in the number of evoked action potentials without changing in the resting membrane potential [143]. Cn2 was used as a selective activator of Nav1.6 channels present at peripheral terminals in the skin, to measure primary afferent response to mechanical stimulus in skin-saphenous nerve and colon-splanchnic nerve preparations. Application of Cn2 at the distal terminals innervating the skin caused increased responses to mechanical stimulation in A-fibers. Peripheral intra plantar injections of Cn2 in mice induces an immediate painful behavior (characterized by lifts, licks, shakes and flinches of the hind paw) lasting for 30 minutes along with the development of a mechanical allodynia [143]. A model of cold allodynia, induced by *ipl* oxaliplatin injections in mice shows a crucial functional contribution of Nav1.6. In this model, 4-aminopyridine and Cn2 *ipl* injections are able to enhance cold allodynia by combining Nav1.6 activation and Kv inhibition [144]. A single mutation on Cn2 (Cn2E15R) was recently introduced to dissociate excitatory and depressant activities, resulting in a Nav1.6 inhibitor able to induce analgesic effects in rodents [145].

In China, *Buthus martensii* Karsch is widely used in traditional medicine, because its venom contains more than 10 analgesic peptides. BmK AGAP (Antitumor AnalGesic Peptide) another α-toxin isolated from *Buthus martensii*, has analgesic properties in a mice visceral model induced by *ip* acetic acid injections, and in inflammatory pain induced by peripheral injection of formalin [146]. Single and double cystein mutations, aimed at modifying the four-disulfide bonds in BmK AGAP, revealed that they are necessary for analgesic activity. The “core domain” located between the α-helix and two beta sheets is considered to be the analgesic domain of BmK AGAP [147]. Mutation of Trp38, a link between the two active surfaces of the peptide, can critically affect the structural stability of the peptide and also its analgesic properties [34,147]. Many other similar peptides (BmK AGP-SYPU1, BmK AGP-SYPU2) but also the insect selective (BmK IT-AP, BmKITAP3), ANEP toxin and BmK AngM1 display analgesic properties [33,138,148-150]. The exact interaction of these toxins with Nav remains unclear. Site directed mutagenesis revealed the prominent role of some residues (Tyr5 and Tyr42) in BmK AGP-SYPU1 for its analgesic activity and in particular for its interaction with the Nav1.7 channel [149,151] but also the role of Gly residues at the C-terminal end for analgesic properties of BmK AGP-SYPU2 (Zhang 2010 BMB).

It seems difficult to explain the pro-nociceptive effect of a scorpion venom that contains several types of toxins, some of which have pro-algic effects and others that are analgesic. In the case of *Buthus martensii* venom, a recent electrophysiological study comparing the successive and simultaneous application of the two toxins BmK I that is algogenic, and BmK IT2 that is rather antinociceptive, shows that Na currents are increased even in the presence of BmK IT2. This suggests that BmK IT2 increases the pharmacological effect of BmK I, and shows an allosteric interaction between sites 3 and 4 of Nav channels [152]. 

The toxin TsNTxP, a long peptide isolated from *Tityus serrulatus*, presenting structural similarities with α- and β-scorpion neurotoxins, was shown to exert antinociceptive effects in mice thermal and inflammatory pain models. It also shows antiallodynic effects in neuropathic pain models. Although not tested on Nav channels, this peptide reduces the release of glutamate in rodent spinal cord synaptosomes, which is an important neurotransmitter in nociceptive transmission that acts through ionotropic and metabotropic receptors [153].

From the venom of *Buthus occitanus*, a long peptide, BotAF was isolated, whose structure resembles a β-like toxin, but has low activity on TTX-S Nav channels from rat DRG. However, BotAF abolishes acute and inflammatory pain in rodents after peripheral or central administration [154]. This suggests a peripheral and spinal mechanism for this peptide activity whose target is yet unknown. 

Kurtoxin, a 63-amino acid peptide isolated from the venom of the scorpion *Parabuthus transvaalicus* is structurally related to the α-scorpion Nav toxins. Kurtoxin is a relative selective inhibitor for low threshold Cav3.1 (α1G) and Cav3.2 (α1H) calcium channels expressed in heterologous systems. Kurtoxin binds with high affinity (Kd=15nM) on a single site on Cav3 and inhibits almost all the Cav3 current at 350 nM concentration [155]. It acts as a gating modifier by shifting the opening of Cav3 channels to more positive voltages. Kurtoxin induces a slight inhibition, at higher concentrations, on high threshold Cav2.2 currents in the rat sympathetic and thalamic neurons [156]. Kurtoxin, also interacts on Nav channels by slowing both activation and inactivation current kinetics, as do α-type scorpion toxins [155]. However, despite its effects on pain-related channels, its activity has not been tested *in vivo* on pain behavior in animal models.

Inhibition of voltage-gated potassium channels (Kv) contributes to neuronal depolarization increased excitability and action potentials prolongation. Scorpion toxins that block Kv channels act synergistically with Nav α- and β-toxins to promote neuronal hyperactivity. Ts8 (also called TsK2 or TsTxKβ) is a long 60 amino acid peptide purified from *Tityus serrulatus* venom able to inhibit selectively Kv4.2 channels (IC_50_ ~ 300-600 nM) without activity on other Kv nor Nav channels. Peripheral (*ipl*) and central (*it*) injections of Ts8 in mice induces a spontaneous pain behavior and a mechanical hyperalgesia [62]. Kv4.2 channels are expressed in the brain and heart. Their activation mediates transient currents in particular in dorsal horn neurons and contributes to modulate nociceptive responses [157]. 

The peptide BmP01 purified from the venom of *Buthus martensii*, is the first scorpion modulator of TRPV1 channels implicated in acute nociception, inflammation, and thermoregulation. BmP01 is a short peptide (29 amino acids) with 3 disulfide bonds stabilized in an Inhibitory Cystine Knot (ICK; [158]) structural fold whose solution structure was resolved [159]. This peptide is devoid of toxicity in mammals and insects. BmP01 dose-dependently activates TRPV1 similarly to capsaicin with an EC_50_ of 132 µM [160]. It was also found to modulate the activity of voltage-gated potassium channels (Kv) by inhibiting mKv1.3, hKv1.3, and rKv1.1, but not mKv1.1, thus presenting species specificity. It is interesting to note that injection of BmP01 in an acidic solution potentiates the pain response in mice, a fact that can be correlated with the acidic properties of scorpion venom [161]. Thus, BmP01 displays strong pH-dependent activity (low potency at neutral pH) showing that this peptide and protons synergize to enhance TRPV1 currents. Injection of BmP01 in mice evokes acute pain responses that reflects its specific effect on peripheral TRPV1, as this pain sensation disappears in TRPV1-KO mice [160]. In this peptide, a key residue, Lys23, has been shown to interact with Glu649 of TRPV1 [162].

Another peptide active in the same family of TRP channels was isolated recently from the Australian Black Rock scorpion, *Urodacus manicatus*. The “Wasabi Receptor toxin”, WaTx, at low concentration, activates TRPA1 channels by binding to an intracellular site, prolongs its open-state duration, and lowers the calcium permeability, without activity on other TRP channels [163]. Peripheral injection in mice hind paw induces a painful behavior comparable to allyl isothiocyanate, the natural activator of TRPA1, but without neurogenic inflammatory symptoms.

## Spider Toxins Interacting with Pain-Related Ion Channels

Spider venoms that are dangerous to humans have been widely studied over the past 30 years, with some being responsible for bites that trigger immediate and more severe pain than others. Several genera belonging to mygalomorph (*Atrax, Hadronyche, Missulena*, *Heteroscodra, Psalmopoeus*…) and to araneomorph (*Phoneutria*, *Heteropoda)* have processed excitatory venoms containing major neurotoxins that modulate the activity of ion channels in sensory fibers (Figure 2). The focus of recent pain research has been on inhibitory toxins and in particular those with pain-relieving properties.

### Nociceptive spider venoms and toxins

Spider bites are generally painful, with varying sensations ranging from immediate localized pain to more general pain that appears later and may persist for several days. Their venoms are a mixture of toxins acting upon both peripheral and central mechanisms. Their neurotoxins can activate peripheral nociceptive receptors (bradykinin B2, 5-HT4, glutamate NMDA and AMPA, tachykinin NK1 and NK2 receptors), or modulate ion channels (TTX-S Nav, TRPV1 and ASIC). Venom components may also activate central receptors (tachykinin, glutamate and CGRP) that are also involved with the production of inflammatory factors such as cytokines IL-1β, TNF-α and prostanoids [26,36]. A large number of pro-algic spider peptides bind to Nav channels and promote their activation either by slowing down current inactivation kinetics, and/or shifting their voltage dependence properties (Table 2). These toxins interact specifically with paddle motifs that correspond to domains I-III for channel opening and to domain IV for Na^+^ current fast inactivation [164]

**Table 2:** Pro-algic spider toxins, ion channels and pain.

Spider species	Toxin	Ion channel affinity (EC_50_ or IC_50_)	Pain phenotype [references]
*Atrax robustus*	*δ-HXTX-Ar1a*	Nav1.1: 30 nM, *Nav1.2*: 39 nM, Nav1.3: 39 nM, Nav1.6: 91 nM (tc)	Acute pain (*ipl*) [174]
*Heteropoda venatoria*	HpTx1 *κ-SRTX-Hv1a*	Kv4.2: 1 µM, Nav1.7: 0.51 µM, Nav1.9: 0.47 µM (tc)	Mechanical hyperalgesia (*ipl*) [173]
*Heteroscodra maculata*	Hm1a *δ-TRTX-Hm1a*	hNav1.1: 38 nM; *hNav1.2*; hNav1.3 (io)	Acute and mechanical pain (*ipl*) [53]
*Macrothele gigas*	*δ-HXTX-Mg1a*	TTX-S Nav: 46 nM; Nav1.1; Nav1.3; Nav1.6 (io)	ND [176]
*Cyriopagopus schmidti*	DkTx	TRPV1: 0.2 µM (tc)	ND [75]
*Phoneutria nigriventer*	PnTx2-6	Nav1.3: 200 nM (tc)	Mechanical hyperalgesia (*ipl*) [166,168-170]
*Psalmopoeus cambridgei*	Vanillotoxins VaTx1, VaTx2, VaTx3	TRPV1: 0.32-12 µM (tc), *Kv2.1*: 7 µM (tc)	Acute and inflammatory pain (*ipl*) [177]

Spider toxin rational nomenclature adopted by the ArachnoServer Spider Toxin Database [341] is given (in italics) as well as common names. Pain-related ion channel and other high-affinity channels (in italics) are indicated with known affinity values. Pain phenotype induced by toxins in rodent models. HXTX: hexatoxin; SRTX: sparatoxin; TRTX: theraphotoxin; io: injected oocytes; tc: transfected cells; ND: not done.

Among araneomorph spiders, the *Phoneutria* species cause the most painful bites in humans, characterized by intense localized pain with inflammatory manifestations (edema, erythema) and hyperalgesia. The South America *Phoneutria* venoms are rich in peptides (more than 80 have been sequenced and characterized, MW 3500-9000 Da, *www.arachnoserver.org*) acting synergistically to produce neurotoxicity [165]. *Phoneutria* venom was shown to induce nociception by the stimulation of sensory fibers containing various pain-related receptors and ion channels such as kinin B2, 5-HT4 receptors, TRPV1, Nav, or ASIC channels [27]. Among nociceptive toxins present in *Phoneutria nigriventer* venom, PnTx2-6, a 48 amino acid peptide is one of the major lethal peptide following intracerebroventricular (*icv)* injection in mice, [166]. Central toxicity is characterized by neuronal hyperactivity associating priapism, salivation, convulsions, and spastic paralysis [167]. Peripheral injections of PnTx2-6 are known to be responsible for priapism, and have been extensively characterized [168]. PnTx2-6, injected in rat hind paw, also induces nociceptive response, in particular a mechanical hyperalgesia that can be measured by the paw pressure test [169]. The mode of action of PnTx2-6 is similar and common to that of α-and β-type scorpion toxins on Nav channels, since the peptide has been shown to slow down the inactivation of neuronal and muscular Nav currents and to shift the voltage dependence of Na^+^ conductance to negative potentials [166,168,170]. Amazingly, the active core of PnTx2-6 was used to design another 19 amino acid peptide, PnPP-19, with properties, that can be used for the therapeutic benefit of erectile dysfunction, without toxicity nor immunogenicity but rather analgesic properties [171]. It was recently shown that PnPP-19 induces analgesic effects via a direct μ-opioid receptors activation and an indirect calcium conductance inhibition [172].

In *Heteropoda venatoria* venom, the peptide HpTx1 was first identified as an inhibitor of Kv4.2 potassium channels. Unexpectedly, HpTx1 was recently shown to inhibit hNav1.7, without effect on rNav1.8 while it can activate hNav1.9 [173]. Sub-micromolar concentrations of this peptide significantly enhanced hNav1.9 currents and slowed down their inactivation. HpTx1 is able to restore nociception in Nav1.7-KO mice by enhancing the excitability of DRG neurons. The peptide, when injected into the hind paw of mice, triggered nociceptive behaviors and in particular mechanical pain, which did not occur in Nav1.7-KO mice. In brief, HpTx1 causes pain in WT and Nav1.7-KO mice and analgesia in Nav1.9-KO mice, but is ineffective in Nav1.8-KO mice. These contrary effects on channels involved in pain reveal the complexity of pain mechanisms and signaling pathways, which may also vary according to the species under consideration.

The venom of Theraphosids, in particular the famous Australian tarantulas *Atrax* and *Hadronyche* (funnel web spiders, Atracidae) contains several δ-hexatoxins (δ-HXTX) responsible for the serious envenomation leading to an excitatory neurotoxic syndrome sometimes fatal in humans. Most of these δ-HXTXs are 42-44 amino acid peptides arranged in an ICK motif and target Nav channels. They all slow the inactivation kinetics of vertebrate TTX-S and insect Nav currents and bind to the voltage sensor domain IV. Recently, 22 δ-HXTX sequences from Australian spider species have been identified and their potency against human Nav channels evaluated [174]. A Fluorescent Imaging Plate Reader assay was used to determine the ability of δ-HXTX-Ar1a, isolated from *Atrax robustus*, to potentiate heterologously expressed hNav channels. δ-HXTX-Ar1a potentiates currents from the pain-related channels Nav1.1, and Nav1.6 but also Nav1.2 and Nav1.3 with nanomolar affinities (EC_50_: 30-91 nM). Nociception was characterized after the *ipl* injection of δ-HXTX-Ar1a (100 nM, 20 µL) in mice that induced acute nocifensive behavior (flinches of the hind paw) for more than 15 minutes. These results strongly support, in addition to their insecticidal predatory function, a defensive role for Atracidae venoms, since all δ-HXTX activate pain-related Nav channels in vertebrates and are thus able to induce algogenic effects during envenomation. δ-HXTXs have slowly evolved from a common ancestry over the past 150-200 million years and today constitute defensive tools able to inflict pain against vertebrate predators with the exception of cats and dogs that are insensitive to Atracidae venoms [174]. Other δ?HXTX isolated from the Australian *Missulena bradleyi* and from the Japanese *Macrothele gigas* share sequence homologies. In particular, δ-HXTX-Mg1a was shown to slow down TTX-S Nav current inactivation and to specifically activate rat Nav1.1 and Nav1.3 and mouse Nav1.6 [175,176]. 

Two peptides, Hm1a and Hm1b (34-35 amino acid), isolated from the Theraphosid *Heteroscodra maculata* were shown to selectively interact with hNav1.1 by slowing down current inactivation. Hm1a significantly prolongs action potential and does not alter the resting membrane potential in rodent central neurons [53]. Chimeric constructs between hNav1.1 and rKv4.1 channels helped to show that Hm1a interacts with both S3b-S4 and the S1-S2 loop of domain IV on sodium channels. Moreover, peripheral injections of Hm1a in mice elicit rapid and intense nocifensive responses. It also produces a strong bilateral sensitization to mechanical stimulation, revealing the role of Nav1.1 in mechanonociception, in particular inducing irritable bowel syndrome, a model of chronic mechanical hypersensitivity.

Although a large number of δ-toxin homologous structures are now known and we can predict their effects on Nav channels, these toxins have not been systematically tested on pain channels or *in vivo* pain models [176]. Other δ-HXTX or δ-TRTX responsible for nocifensive effects are likely to emerge in the coming years.

Vanillotoxins (VaTx1, VaTx2 and VaTx3) are 34-35 amino acid peptides with ICK fold, purified from *Psalmopoeus cambridgei* venom, able to activate TRPV1 channels with affinities ranging from 0.3 to 12 µM [177]. Vanillotoxins seem specific for TRPV1 since they do not activate other pain-related TRPA1 nor TRPM8 channels. They interact with the outer pore region of TRPV1, on important sites within S5-S6 segments [75]. VaTx1 also blocks with the same affinity Kv2.1 channels in a voltage-dependent manner. Intra plantar injection of VaTx3, the most potent toxin on TRPV1, produces painful symptoms in mice characterized by licking and flinching, thus reproducing the equivalent behavior as with crude venom injection.

Another spider peptide, DkTx, is able to selectively and irreversibly activate TRPV1, without effect on other pain-related ion channels [75]. DkTx, purified from *Cyriopagopus schmidti* (former *Ornithoctonus huwena*) venom, has a very original structure since it is composed of two independently folded ICK peptides connected by a 7 residue linker, which explains the name “double-knot toxin” (DkTx). The single knot peptides, tested alone on TRPV1 were 5 to 50 fold less active. DkTx binds on TRPV1 in an open state, and in particular within the pore domain where several interaction sites in S5-S6 are important for its effects. The structure of DkTx has been solved together with its unusual mechanism of activation on TRPV1. This revealed an important interaction of the toxin within the surrounding lipid membrane that stabilizes the toxin-channel complex [178]. The two ICK knots of DkTx help to prolong the lifetime of the complex, and the toxin has a small protein-protein interface with TRPV1. A disruption of hydrophobic residues behind the selectivity filter leads to the opening of the channel. rTRPV1 channels have been functionally expressed in worms’ polymodal nociceptive neurons to confer a specific, robust, and dose-dependent avoidance behavior to capsaicin. However, DkTx that also binds to rTRPV1 does not elicit aversive behavior in *C. elegans* even at high concentrations [179]. DkTx activates rTRPV1 but its effects on nociception are still unknown in rodents and further study will be required.

*α-latrotoxin: a particular and unique case*

The black widow spider has become famous throughout the world for the dreaded effects of its venom on humans. Its bite leads to a very painful and long lasting cholinergic syndrome characterized by sustained muscular contractures (with the characteristic facial trismus), nausea, vomiting, dehydration, diarrhea that can lead to dramatic complications such as respiratory paralysis in fragile subjects [180-183]. The toxin responsible for this strong neurotoxicity belongs to a family of 3 proteins of 120-130 kDa called α-latrotoxins (α-LTx), each being specific to crustaceans, insects or mammals [184,185].

By binding selectively and almost irreversibly to two types of receptors at the presynaptic nerve endings, α-LTx causes membrane depolarization that results in a massive influx of Ca^2+^ into the cells. The exocytosis of the synaptic vesicles and the subsequent massive release of neurotransmitters cause a slow and long-lasting contracting paralysis in the mammal or insect victim [185,186]. A neurexin has been identified as the first receptor at the presynaptic level, it binds α-LTx in a calcium-dependent manner and interacts intracellularly with the protein synaptotagmin [187]. A second receptor, called CIRL-latrophilin (or CL1) binds α-LTx in a calcium-independent way [188]. In a normal situation, synaptotagmin exerts a negative control on the release of neurotransmitters. α-LTx, after binding to its receptor, inserts itself into the plasma membrane to form non-selective cation channels [189]. These high conductance channels, which are permanently opened, allow the Ca^2+^ entry responsible for the fusion of vesicles and the release of neurotransmitters [190]. The mode of action of α-LTx would thus be to lift, via its receptor, the inhibitory control of synaptotagmin, thanks to the entry of Ca^2+^ ions. However, even in the absence of its receptor, it seems that α-LTx can form pores in membranes by inserting itself into the lipid bilayer [191]. The three-dimensional structure of α-LTx determined by cryo-electron microscopy shows that this protein can exist in two forms: dimeric or tetrameric. The tetrameric form represents the active form of α-LTx, able of inserting itself into the lipid bilayer to form cationic channels [188,192]. The interaction with the cell surface would be via the N-terminal domain of each monomer. 

The α-LTx case illustrates the extraordinary capacity and plasticity of neurotoxins to interact, in a specific way and with a wide variety of mechanisms, with neuronal plasma membrane to create ion flows and modulate, or even exacerbate, a physiological response (here the sustained muscle contractions and cramps) contributing to accentuate the pain felt by a prey or an aggressor.

### Interaction of spider toxins with ion channels and analgesic properties

Antinociceptive spider toxins and toxin blocking pain-related ion channels are presented in Table 3. Spider genus and species are listed according to accepted taxonomy (Web Spider Catalog: https://wsc.nmbe.ch/). Spider toxin rational nomenclature adopted by the ArachnoServer Spider Toxin Database [341] is given (in italics) as well as common names.

**Table 3.** Spider toxins, ion channels and antinociceptive effects.

Spider species	Toxin rational nomenclature	Ion channel: affinity (IC_50_, EC_50_, Kd)	Analgesia (phenotype) [references]
*Agelenopsis aperta*	AG489, AG505	TRPV1, 300 nM (io)	ND [292]
	*ω-AGTX-Aa2a* ω-Aga-IIA	Cav2.1, cCav2.2: 1-14 nM	ND [272]
	*ω-AGTX-Aa3a* ω-Aga-IIIA	Cav2.1: 0.5 nM (nn), Cav2.2: 1.5 nM (nn), Cav1: 1 nM (nn)	ND [273,342,343]
	*ω-AGTX-Aa4a* ω-aga-IVA	Cav2.1: 2-6 nM (nn)	Mechanical and heat hyperalgesia in inflammatory pain [268-271]
*Ceratogyrus marshalli (cornuatus)*	*β-TRTX-Cm1a* CcoTx1	Nav1.1: 500 nM, *Nav1.2*: 3 nM, Nav1.5: 323 nM, Nav1.8: 2 µM (io)	ND [245]
*Ceratogyrus darlingi*	*β-TRTX-Cd1a* Cd1a	hNav1.7: 16 nM (tc), hNav1.8, rCav2.2: 3 µM (tc)	Acute pain [242]
*Chilobrachys* *jingzhao*	JZTX-34	TTX-S Nav: 85 nM, Nav1.7: 610 nM (tc)	Acute, heat and inflammatory pain [211,212]
	*β-TRTX-Gj2a* JZTX-V	rNav1.7: 62 nM (tc), rNav1.4: 5 nM (tc), hNav1.7: 0.63 nM (tc), Kv4.2: 604 nM (io)	ND [207]
	5Br-Trp24-JZTX-V (AM6120)	hNav1.7: 0.8 nM (tc), mNav1.7: 5.4 nM (tc), *hNav1.4:*104 nM (tc)	Anti-pruritis [210]
*Cyriopagopus albostriatus*	µ-TRTX-Ca1a Ca1a	Nav1.7: 378 nM, Nav1.6: 547 nM, *Nav1.2*: 728 nM (tc)	Acute, heat and inflammatory pain [215]
	µ-TRTX-Ca2a Ca2a	Nav1.7: 98 nM, *Nav1.2*: 216 nM, Nav1.6: 313 nM, Nav1.3: 491 nM (tc)	Acute, heat and inflammatory pain [214]
*Cyriopagopus doriae*	*µ-TRTX-Hd1a* Hd1a	hNav1.7: 111 nM (io), hNav1.1 (io)	ND [195]
*Cyriopagopus hainanus*	μ*-TRTX-Hhn2a* HainanTxIII	Nav TTX-S: 1 nM (nn), hNav1.7: 211 nM, hNav1.3: 491 nM (tc)	Inflammatory, visceral and neuropathic pain [231,344]
	*µ-TRTX-Hhn1b* HainanTxIV (*β-TRTX-Hn2a*)	TTX-S Nav: 45 nM (nn), hNav1.7: 21 nM, *Nav1.2*: 31 nM, Nav1.3: 375 nM (tc)	Inflammatory, visceral & neuropathic pain [228,344,345]
*Cyriopagopus schmidti*	*µ-TRTX-Hs1a* Hs1a	Nav1.1: 19.4 nM, Nav1.6: 19.2 nM, Nav1.7: 27 nM (tc)	ND [201]
	*µ/ω-TRTX-Hh1a* HWTx-I	Cav2.2: 100 nM (nn), TTX-S Nav: 55 nM (nn)	Inflammatory pain [278,346,347]
	μ*-TRTX-Hh2a* HWTX-IV	hNav1.7: 17-100 nM (tc), hNav1.6: 52 nM (tc), *hNav1.2*: 44-150 nM (tc), hNav1.1: 41 nM (tc), hNav1.3: 350 nM (tc)	Inflammatory, visceral and neuropathic pain [221,222,227,236,348]
	*ω-TRTX-Cs-10* HWTX-X	Cav2.2: 40 nM (nn)	ND [280]
	*ω-TRTX-Cs-16* HWTX-XVI	Cav2.2: 60nM (nn)	Heat, inflammatory and post-operative pain [281]
*Cyriopagopus lividus*	*µ-TRTX-Hl1a* Hl1a	Nav1.8: 2 µM (tc)	Inflammatory, visceral and neuropathic pain [244]
*Cyriopagopus schioedtei*	*µ-TRTX-Cs1a* Cyriotoxin1a	TTX-S Nav: 170 nM, hNav1.1: 72 nM, *hNav1.2*: 76 nM, hNav1.6: 115 nM, hNav1.7: 130 nM(tc)	Heat and mechanical pain [213]
*Cyriopagopus* sp. *(Phlogiellus)*	*µ-TRTX-Phlo1a* PhlTx1	hNav1.7: 39-260 nM (tc-io)	Acute and inflammatory pain [204,232]
*Davus fasciatus*	*µ-TRTX-Df1a* Df1a	hNav1.7: 2 nM, hNav1.3: 3 nM, hNav1.6: 8 nM, hNav1.1: 14 nM, hCav3.2: 253 nM (tc)	Acute pain [241]
*Geolycosa* sp.	Purotoxin (PT1)	P2X3: 12 nM (nn)	Heat hyperalgesia in inflammatory pain [301]
*Grammostola porteri (G. rosea)*	*µ-TRTX-Gp1a* GpTxI	Nav1.7: 4.4-10 nM, Nav1.3: 20 nM	Acute, thermal, inflammatory, visceral pain Neuropathic (mechanical and thermal hyperalgesia) pain [218,219,333]
*Grammostola rosea*	*ω-TRTX-Gr1a* ω-grammoTx-SIA	Cav2.1, Cav2.2 (nn)	ND [343,349,350]
	*M-TRTX-Gr1a* GsMTx4	Piezo1: 155 nM, Piezo2: < 5 µM (tc) TRPC1: < 5 µM, TRPC6: < 5 µM, TRPA1: 1µM (tc)	Mechanical inflammatory and neuropathic pain [293-297,302,303,351]
	*β-TRTX-Gr1b* GsAF-I	hNav1.7: 1-40 nM (tc)	ND [203,204]
	*β-TRTX-Gr2c* GsAFII	hNav1.7: 13.6-1000 nM (tc)	ND [203,204]
	*β-TRTX-Gr1a* GrTx1	hNav1.7: 15-370 nM (tc)	ND [203,204]
*Hadronyche infensa*	π-*TRTX-Hi1a* Hi1a	rASIC1a: 0.4 nM, hASIC1a: 0.52 nM (tc)	ND [291]
*Heteropoda venatoria*	*K/δ-HPTX-Hv3a* HpTx3	Nav1.7 : 136 nM (tc); Kv4.2: < 100 nM (io)	Acute, inflammatory, and neuropathic pain [35]
*Heteroscodra maculata*	π-*TRTX-Hm3a* Hm3a	rASIC1a: 1-2 nM (io), hASIC1a: 40 nM (io), rASIC1b: 46.5 nM (io), hASIC1b: 178 nM (io)	ND [290]
*Hysterocrates gigas*	*ω-TRTX-Hg1a* SNX482	Cav2.3: 15-30 nM (tc), Kv4.2: < 3 nM (tc)	Neuropathic pain [69,275,276]
*Nephila clavata*	JSTX	AMPA ionotropic R: < 500 nM (nn)	Thermal hyperalgesia, mechanical allodynia [306,307]
*Pamphobeteus nigricolor*	*µ-TRTX-Pn3a* Pn3a	Nav1.7: 0.9 nM-1457 nM (tc)	Acute pain Inflammatory pain (with opioid co-administration) [204,233]
*Phoneutria nigriventer*	*δ-CNTX-Pn1a* PnTx4(6-1)	Insect Nav, mam. CB1& opioid R	Acute , inflammatory and neuropathic pain [205,206]
	PnTx1	*Nav1.2*: 34 nM, Nav 1.7: < 1 µM, Nav1.4, Nav1.3 (io)	ND [197,352]
	*ω-CNTX-Pn2a* PnTx3-3	Cav2.1: < 60 nM (nn), Cav2.2: < 10 nM (nn) Cav2.3: 12 nM (nn)	Acute thermal pain, neuropathic mechanical allodynia [247-251]
	*ω-CNTX-Pn3a* PnTx3-4	Cav2.1: 50 nM (tc), Cav2.2: 50 nM (tc)	Inflammatory and post-operative pain [252,253]
	*ω-CNTX-Pn4a* Phα1β PnTx3-6	Cav2.2: 122 nM (tc), Cav2.3: 136 nM (tc), Cav2.1: 263 nM (tc), rTRPA1: 32 nM (nn), hTRPA1: 681 nM (tc)	Acute, inflammatory, neuropathic and post-operative pain [254,256,259,261-264,267]
	*U7-CNTX-Pn1a* PnTx3-5	TRPV1: 45 nM (tc)	Acute, post-operative, and neuropathic pain [298,299]
	Γ-CNTX-Pn1a PnTx4(5-5)	NMDA-R: < 100 nM (nn)	Hyperalgesia on inflammatory pain [308,309]
*Phormingochilus everetti*	*β/µ-TRTX-Pe1b*	Nav1.7: 167 nM (io)	ND [199]
*Phrixotrichus auratus*	*κ-TRTX-Gr4a* VSTx-3	Nav1.7, Nav1.3, Nav1.8 (tc)	ND [198]
*Psalmopoeus cambridgei*	*π-TRTX-Pc1a* PcTx1	rASIC1a: 0.4-3.7 nM, rASIC1a+2b: 3 nM; rASIC1b: 100 nM (io, tc)	Acute, thermal, inflammatory, mechanical and neuropathic pain [76,284]
*Psalmopoeus reduncus*	*β/δ-TRTX-Pre1a*	Nav1.1: 57 nM; Nav 1.2: 190 nM, Nav 1.6: 222 nM, Nav 1.7: 15-114 nM (io, tc)	ND [200]
*Theraphosa apophysis*	*TRTX-Tap1a* Tap1a	hNav1.1: 301 nM, *hNav1.2*: 95 nM, hNav1.3: 179 nM, hNav1.6: 191 nM, Nav1.7: 80 nM, Cav3.2: 1.23 µM (tc)	Mechanical hyperalgesia in visceral pain [243]
*Trixopelma pruriens*	*β/ω-TRTX-Tp1a* ProTx-I	*Nav1.2*: 104 nM; Nav1.6: 21 nM, Nav1.7: 51-95 nM, Nav1.8, Nav1.5: 20 -358 nM rCav3.1: 50 nM, hCav3.1: 640 nM (tc), Kv2.1: 411 nM, hTRPA1: 389 nM (tc)	ND [199,234,235,300,353]
	*β/ω-TRTX-Tp2a* ProTx-II	Nav1.7: 0.3-72 nM, hNav1.1: 16 nM>, hNav1.3: 25 nM, *Nav1.5: 19-400 nM*, hNav1.6: 31 nM, Cav3.1: 150 nM, hCav3.2: > 1 µM (tc)	Acute and inflammatory pain [37,234,235,237,238,353]
	*µ-TRTX-Tp1a* ProTx-III	hNav1.7: 2-11 nM (tc), hNav1.1: 11.3 nM, hNav1.3: 11.5 nM, hNav1.6: 0.29 µM (fi)	ND [239]

AGTX: agelenatoxin; CNTX: ctenitoxin; TRTX: theraphotoxin, HPTX: heteropodatoxin. Pain-related ion channel and other high-affinity channels (in italics) are indicated with known affinity values (IC_50_, EC_50_, Kd). Io: injected oocytes; tc: transfected cells; nn: native neuronal currents; fi: fluorescent imaging; ND: not done. Type of analgesia induced by toxins and reported from rodent model experiments. *Cyriopagopus (*former *Haplopelma) doriae; Cyriopagopus hainanus (Ornithoctonus* or *Haplopelma* or *Selenocosmia hainana); Cyriopagopus schmidti (*former *Haplopelma* or *Ornithoctonus huwena); Chilobrachys jingzhao (*former *C. guangxiensis)*; *Grammostola rosea (*former *G. spatulata); Phrixotrichus auratus (*former *Paraphysa scrofa).*

*Inhibition of Nav channels*

Curiously, while spider bites are quite painful, a very large number of analgesic toxins were identified, which block the activity of Nav involved in pain. The search for specific inhibitors of the Nav1.7 channel, one of the major actors in the transmission of pain in the PNS, has led to the identification from tarantula venoms of more than 20 peptides having between 26 and 35 amino acids and belonging to different structural families. Off-targets activities, especially on the skeletal muscle isoform Nav1.4, on the cardiac isoform Nav1.5, but also on neuronal isoforms Nav1.2 and Nav1.6, could impact the therapeutic window and cause dose-limiting adverse effects [193].

Twelve families of peptides that target Nav and called “NaSpTx” (for voltage-gated sodium channel spider toxin) families 1-12, have been defined based on their activity or sequence similarity [194]. Families NaSpTx1-3 include toxins that are more or less potent and selective for pain-related Nav channels. In the NaSpTx family 1, all peptides were isolated from the venom of tarantulas (Theraphosidae), and consist of 33-35 amino acid residues with 3 disulfide bridges that form an ICK motif. They bind to the S1-S2 and S3-S4 linkers of Nav domain II (neurotoxin site 4). In the NaSpTx2 family, more than 30 peptides with 33-41 residues and ICK motif inhibit Nav, Cav or Kv channels. They bind to S3-S4 linker of Nav domain IV (site 3). NaSpTx3 family includes peptides with 29-32 residues, 3 disulfide bonds, ICK motif, which act as gating modifiers by binding to the voltage sensor region (S3-S4 linker) of channel domain II (site 4). 

Some peptides block pain-related Nav but have not yet been tested on nociceptive models, which does not allow their analgesic function to be asserted. This is the case for several theraphosids peptides, such as μ-TRTX-Hd1a from *Cyriopagopus* (former *Haplopelma*) *doriae*, μ-TRTX-Ccy1a, -1b from *Chromatopelma cyaneopubescens*, μ-TRTX-Osp1a, -1b from *Orphnaecus* sp., μ-TRTX-Ep1a from *Cyclosternum* (former *Euathlus*) *pulcherrimaklaasi,* μ-TRTX-Phlo1a, -Phlo1b, from *Cyriopagopus* sp*.* (former *Phlogius)*, and μ-TRTX-Se1a from *Selenocosmia effera* venoms, which inhibit human Nav1.7. All have 32-35 amino acids, an ICK structure, and are members of NaSpTx-F1-F2 or -F3 [195-197]. A high level of selectivity was found for μ-TRTX-Hd1a, which inhibits hNav1.7 with a good affinity (IC_50_ 111nM) but also Nav1.1 [195]. 

Many other spider peptides can be listed with such inhibitory effects on pain-related Nav channels. The voltage sensor toxin 3 (VSTx-3 or κ-TRTX-Gr4a), a 34 amino acid peptide purified from *Grammostola rosea* venom, is a Cav and Kv blocker but also an inhibitor of TTX-S hNav1.3, hNav1.7 and Nav1.8. [198]. From the same venom, GTx1-15 peptide potently inhibits hNav1.7 (IC_50_ 7nM) and Nav1.3 with minor effects on TTX-R channels. The two peptides (34 residues) β/μ-TRTX-Pe1a and β/μ-TRTX-Pe1b from the arboreal tarantula *Phormingochilus everetti* are Nav1.7 inhibitors. The recombinant Pe1b peptide shows potent and relative selective inhibition (IC_50_ 167nM) on Nav1.7 compared to other Nav isoforms such as Nav1.6, Nav1.5 and Nav1.2 [199]. 

The β/δ-TRTX-Pre1a is a 35 residue peptide, isolated from the tarantula *Psalmopoeus reduncus*, which inhibits activation of hNav1.2, hNav1.6, and hNav1.7, and both activation and inactivation of Nav1.1. The β/δ-TRTX-Pre1a interact with the S3-S4 loops in DII and DIV Nav channels voltage sensors and with some residues in the S1-S2 loops that are critical for imparting isoform selectivity [200].

The Hs1a peptide isolated from the Chinese tarantula *Cyriopagopus* (former *Haplopelma*) *schmidti* is a potent blocker of the pain-related channels hNav1.1, hNav1.6, and hNav1.7 and others such as hNav1.2 and hNav1.3. A fluorescent recombinant peptide, Hs1a-FL was designed as a vector for delivering an optical sensor to target Nav1.7 in the peripheral nerves of mice *in vivo* [201]. HS1a-FL was hence developed as a near-infrared imaging agent used to visualize the limits of an operating area during surgery around nerves and to avoid nerve damage with serious or irreversible consequences.

PnTx1 is a longer peptide with 78 residues, isolated from the Ctenidae *Phoneutria nigriventer*, which inhibits rat Nav1.7 but also Nav1.2, Nav1.3 and Nav1.4 with comparable affinities and has no effect on hNav1.5 nor insect Nav [197]. It is known to induce neurotoxicity after *icv* injection in mice but its peripheral effects have not been yet described.

For most of these peptides, effects on rodent pain model have not yet been investigated, probably because of a lack of selectivity on pain-related channels. Another limiting factor in the search for new analgesics is the target promiscuity of spider peptides. Studies showed that several *Grammostola* toxins are active on Kv, Cav and Nav channels, and some of them can act both as pore-blockers and gating-modifiers on different ion channels [202]. As an example, the peptide GsAFI has inhibitory effects on several Nav channels (IC_50_ 1-40 nM on Nav1.7) but also on the cardiac potassium channel hERG1 at higher concentrations that makes it unusable for the design of pain medication [203,204].

*Analgesic peptides that target Nav channels*

One of the most painful venoms among araneomorph spiders is that of the *Phoneutria* species, which is however composed of several analgesic peptide toxins. The δ-CNTX-Pn1a (PnTx4(6-1) is a 48 amino acid peptide presenting high toxicity to insects but not to rodents after central injections. This α-like toxin targets insect Nav without affecting mammalian Nav channels[205]. δ-CNTX-Pn1a (5µg, *ipl*) induces analgesic effects in several pain models in rats including carrageenan-induced inflammatory hyperalgesia [206]. In a neuropathic (SNI) pain model, δ-CNTX-Pn1a (0.5µg, *it*) rapidly reverses hyperalgesia induced by sciatic nerve constriction. δ-CNTX-Pn1a (0.5 µg, *it*) also induces antinociceptive effects on an acute pain model (prostaglandin E2, *ipl*). Although the direct involvement of ion channels in this analgesic effect is not known, it was shown that other receptors are involved in the opioid and cannabinoid endogenous systems [206]. Broader research would make it possible to identify more specific targets among other mammalian ion channels.

The venom from the large araneomorph *Heteropoda venatoria* contains the HpTx3 peptide, a Kv4.2 potassium channel inhibitor, which was recently found to be a potent and selective hNav1.7 blocker (IC_50_ 136 nM) [35]. Its interaction in S3-S4 loop in domains II and IV of Nav is representative of a mixt pore blocking and gating modifier effect. When HpTx3 (0.2-5 mg/kg) is peripherally injected in mice, analgesic effects are observed in acute (acetic acid, hot plate), inflammatory (formalin, CFA) and neuropathic (SNI) pain models [35].

Two analgesic peptides have been isolated from the Chinese earth tiger *Chilobrachys jingzhao* venom: JZTX-V, 29 residues, which inhibits TTX-R and TTX-S Nav with potent affinities (IC_50_ around 30 nM) in rat sensory neurons [207]. It alters the gating properties of channels, by shifting Na^+^ current activation and inactivation curves. A complete blockade of the skeletal muscle rNav1.4 is observed with JZTX-V, while the inhibition of hNav1.7 is incomplete, which could lead to adverse effects [208]. The search for selective inhibitors for Nav1.7 led to the mutation of a key residue, Ile28, which contributes to the affinity of JZTX-V for Nav1.4. Several analogues, with the Ile28Glu mutation, present a higher affinity and selectivity for TTX-S Nav over TTX-R Nav in DRG neurons and inhibit Nav1.7 with sub-nanomolar affinities [209]. Other mutations leading to 5Br-Trp24-AM-6120 peptide provide optimization of selectivity and potency for hNav1.7 and mNav1.7. This latter mutant also showed analgesic properties following 2 mg/kg *sc* injections, on a mice pruritis model induced by histamine [210]. In the same venom, the peptide JZTX-34, blocks TTX-S Nav (IC_50_ 85 nM) in rat DRG neurons and shifts the steady state inactivation curve, without effect on TTX-R Nav [211]. JZTX-34 preferentially blocks Nav1.7 (IC_50_ 610 nM) compared to other Nav1 isoforms, by binding to the S3-S4 linker on domain II voltage sensor, in particular on a critical residue (D816) and trapping domain II S4 in a resting state [212]. JZTX-34 (0.5 to 2 mg/kg, *ip*) dose-dependently reverses acute and inflammatory pain in the formalin test in mice. JZTX-34 also reduces abdominal contractions and hind limb movements in the acetic acid-induced writhing test. In the hot plate test, JZTX-34 (*ip*) increases the latency time for escaping [212].

The venom of the Malaysian earth tiger *Cyriopagopus* species contains analgesic peptides like Cyriotoxin 1a (CyrTx-1a, 33 amino acids, NaSpTx1 family) isolated from *C. schioedtei*. CyrTx-1a inhibits human Nav1.1, Nav1.3, Nav1.6 and Nav1.7 with nanomolar affinities and minor effects on Cav or Kv channels. The peptide was selected, after a high throughput screening of 117 venoms tested on functional automated patch‐clamp assays, for their ability to block hNav1.7 with high potency and selectivity [213]. CyrTx-1a also inhibits TTX-S Nav currents in mouse DRG neurons (IC_50_ 170 nM) with a good selectivity over TTX-R Nav. Despites its effects on Nav1.6, *ipl* injections of the peptide does not impair mice skeletal neuromuscular excitability. Analgesic properties of CyrTx-1a (102 nmol/kg, *ipl*) were evaluated on a hot plate model. It is important to note that *in vivo* toxicity (50% death) was seen using quite the same concentrations (144nmol/kg), but after intra muscular injections.

*Cyriopagopus albostriatus* venom contains two peptides, μ-TRTX-Ca1a and μ-TRTX-Ca2a, 38 and 35 amino acid respectively, which are more selective for hNav1.7 and do not alter its voltage-dependent properties [214,215]. Ca1a (100-500µg/kg, *ipl*) and Ca2a (50-200 µg/kg, *ip* or *ipl*) show analgesic properties in acute (acid acetic-induced writhing), inflammatory (formalin test) and heat (hot plate) pain models in mice, dose-dependently attenuating pain behaviors [214,215].

In *Grammostola porteri* venom, GpTx1 (also called Gtx1-15; 34 residues, NaSpTx1 family [198]), has a potent inhibitory activity on Nav1.7 (IC_50_ 10 nM) and a good selectivity over Nav subtypes [216]. Different amino acid substitutions (Ala, Glu, Arg and Lys scans) were designed to obtain mutant peptides with single digit nanomolar potency on Nav1.7 and more selectivity against Nav1.4 and Nav1.5 [216,217]. The co-injection of GpTx1 (30-300nM, *ipl*) with the painful scorpion peptide OD1, dose-dependently reduces spontaneous pain behavior in mice. However, a systemic injection (*ip*) of GpTx1 is unable to reverse OD1 effects even at a high concentration (0.1 mg/kg) [218] and induces motor deficits (ataxia) at higher doses (0.3 mg/kg), which is probably due to its inhibitory effect on Nav1.6. A higher selectivity for Nav1.7 over Nav1.6 seems necessary to get an acceptable therapeutic window for systemic use of analgesic peptides. In other pain models, the central (*it*) injection of GpTx1 induces antinociception involving the opioid system but without tolerance nor motor side effects [219]. GpTx1 (0.05-5 pmol, *it*) dose-dependently produces analgesia in the tail flick test and in the acetic acid-induced visceral pain. Gptx1 (0.1-5 pmol, *it*) also prevents mechanical allodynia in carrageenan-induced inflammatory pain, and thermal hyperalgesia in CCI-induced neuropathic pain [219]. Moreover, GpTx1 (0.1-10 pmol, *it*) reduces the nociceptive acute and inflammatory responses induced by formalin injection. 

Venoms from the Chinese bird-eating *Cyriopagopus* sp*. (formerly Ornithoctonus or Selenocosmia or Haplopelma)* are rich in peptides modulating Nav channels. Huwentoxin IV (HWTX-IV, 35 amino acid, NaSpTx1 family) a peptide isolated from *Cyriopagopus schmidti* specifically inhibits TTX-S Na^+^ currents without effect on TTX-R Na^+^ currents in rat DRG neurons [220]. HWTX-IV potently inhibits hNav1.7, rNav1.2, rNav1.3 and hNav1.6 [221,222]. It has no effects on skeletal muscle rNav1.4 and cardiac hNav1.5. HWTX-IV binds to site 4, in particular with 5 residues that are crucial to impair activation of Nav1.7, trapping the domain II sensor in a closed state [223]. A docking model of the toxin on Nav1.7 has been proposed showing that several key molecular determinants forming a hydrophobic patch are important for its interaction [224,225]. In order to find new powerful analgesics, several HWTX-IV analogs were synthesized showing an increased potency and/or selectivity on hNav1.7 over other Nav isoforms [221,226]. HWTX-IV induces analgesic effects in inflammatory and neuropathic pain models in rodents [227]. Injection of HWTX-IV (100 µg/kg) in rats produces an efficient reversal of formalin-induced hyperalgesia and a protective effect after acetic acid injection. Moreover, HWTX-IV induces antiallodynic effects on a spinal nerve model of chronic neuropathy. However, the interaction of HWTX-IV with Nav1.6 may suggest *in vivo* motor side effects that could impair its use as a therapeutic analgesic drug [222].

The venom of the tarantula *Cyriopagopus* (former *Haplopelma*) *hainana* contains two main peptides, Hainantoxin-III and -IV, presenting sequence homologies with HWTX-IV. HNTX-III and HNTX-IV inhibit TTX-S Nav currents in rat DRG (IC_50_ 1.1 and 44.6 nM respectively) and do not affect Cav nor Kv channels [228]. These peptides shift the Na^+^ current inactivation curve in the hyperpolarizing direction without affecting activation and inactivation kinetics. In particular, they inhibit hNav1.7 current activation in a manner similar to HWTX-IV, trapping the voltage sensor in the closed state [229]. HNTX-III and HNTX-IV bind to site 4, located on the extracellular S3-S4 linker of hNav1.7 domain II [230]. Both peptides (50 to 200 µg/kg, *ip* pre-treatment) produce analgesia in inflammatory (CFA, formalin), acute visceral (acetic acid injections) and neuropathic (SNI) pain models in rodent, by reversion of mechanical hyperalgesia, without CNS side effects [227,231].

Phlotoxin-1 (PhlTx1, a 34 amino acid) isolated from *Cyriopagopus* (former *Phlogiellus*) sp., presents low sequence homologies with the other identified peptides in the NaSpTx1 family. PhlTx1 inhibits hNav1.7 with potent affinity (IC_50_ 39 nM) and selectivity, with almost irreversible effects. It slightly inhibits hNav1.4 and hNav1.6 at high (1µM) concentration [204,232]. The blocking efficiency of PhlTx1 was compared with that of 10 leading spider toxins, belonging to NaSpTx1-3 families, known to inhibit Nav1.7, in the same experimental conditions [204]. The most potent hNav1.7 inhibitor peptides in this study are HNTX-IV, HWTX-IV, HWTX-I, ProTx-I, ProTx-II, GsAFI, GsAFII, GrTx1 and PhlTx1 with IC_50_ below 50 nM. Injections of PhlTx1 (0.47µg, *it*) reduces acute and inflammatory pain in the OD1 and formalin models in mice without neurotoxic effects [232].

The peptide μ-TRTX-Pn3a (Pn3a, 35 amino acids) isolated from *Pamphobeteus nigricolor* potently inhibits hNav1.7 (IC_50_ 0.9 nM) with an exquisite selectivity over all hNav isoforms, Kv, Cav channels and nicotinic acetylcholine receptors [233]. Pn3a is a gating modifier toxin that shifts the voltage dependence of Nav1.7 activation to more depolarized membrane potentials. It binds to S3-S4 linkers in the voltage sensing DII and DIV domains of Nav1.7. Pn3a at the highest dose tested (3 mg/kg, *ip*), induces a sustained analgesic activity, on acute (OD1, *ipl*) spontaneous pain model. Curiously, no attenuation of noxious heat pain was observed on the hot plate model. Moreover, the peptide was devoid of anti-allodynic activity in inflammatory pain models in rodents when administered systemically except when it was co-injected with subtherapeutic doses of opioids [233]. More recent studies have shown that Pn3a is a poor inhibitor for hNav1.7 (IC_50_>1µM) in mammalian transfected cells that raises the question of its real affinity for Nav1.7 [204].

The protoxins, ProTx-I (35 amino acids, NaSpTx2 family) and ProTx-II (30 amino acids, NaSpTx3 family) are ICK peptides isolated from *Trixopelma pruriens* venom. They are the first inhibitors for the TTX-R Nav, by blocking hNav1.5 and rNav1.8 currents with a similar potency (IC_50_ 20-30nM), acting like gating modifiers [234,235]. They are also potent inhibitors of the TTX-S Nav1.2, Nav1.6, and Nav1.7. ProTx-II is more potent on Nav1.7 than on Nav1.5, and inhibits the fast inactivation of hNav1.7 by trapping the domain IV voltage sensor in the resting conformation [236]. ProTx-I and -II also shift the voltage dependence of activation of Cav3.1 [234,235]. Moreover, sub-micromolar concentration of ProTx-I inhibits the potassium Kv2.1 channel and TRPA1 that represent an important off target impact. On skin-nerve preparation, ProTx-II (0.3-10 µM) is able to reduce the amplitude of C-fiber action potential with weaker effects on Aβ-fibers, only on desheathed sensory nerves [237]. The maximum tolerated dose is 2 µg (*it*) in rats, since higher doses induce weakness, hind and forelimbs paralysis, and death. The motor effects probably result from the inhibition of other Nav isoforms such as Nav1.1 and Nav1.6. However, weak doses of ProTx-II (1-2 µg) induce analgesic effects in thermal pain models as well as in the formalin-induced acute and inflammatory pain [37]. ProTx-II was also shown to control pain in burn injury by reducing the frequency of excitatory post synaptic currents in spinal dorsal horn neurons [238].

ProTx-III, also called Tp1a (33 amino acids), isolated from *Trixopelma pruriens,* preferentially inhibits the hNav1.7, hNav1.6, hNav1.1 and hNav1.3 isoforms [239]. ProTx-III potently inhibits Nav1.7 (IC_50_ 2 nM) and does not affect other Cav channels involved in pain pathways. The antinociceptive effect of ProTx-III (12 and 40 pmol, *sc*) was observed by reduced flinching behavior in a model of spontaneous pain induced by OD1 in mice [239].

ProTx analogues were designed to improve selectivity for Nav1.7 based on peptide pharmacophore and channel interaction [199]. Several mutants, with modified C terminus (ProTx-I-NH2 and ProTx-II-NHCH3), have increased activity on both Nav1.2 and Nav1.7 and a decreased activity on Nav1.5 and Nav1.6 [240]. It seems that point modifications of amino acids on similar pharmacophores including the C-term residue, can affect the affinities of toxins for Nav channels. This shows the real difficulty in obtaining a selective toxin for Nav1.7 without affecting its effect on other Nav channels.

ProTx-II was used as a scaffold to design JNJ63955918, a longer peptide with two mutations (W7Q, W30L) providing improved Nav1.7 selectivity and *in-vivo* tolerability [37]. This mutant binds to the closed state of Nav1.7 and prevents its activation in a voltage insensitive manner. JNJ63955918 induces an insensitivity to pain, in rat models of thermal and chemical nociception, lasting for 6 h after *it* injection. Doses up to 5 µg/10 µL are well tolerated with no detectable severe adverse effects except itching behavior. The analgesic properties of JNJ63955918 were also proved in tail flick and hotplate pain models, by a continuous *it* infusion (0.5 µg/h) in rats during 14 days without serious adverse effects. JNJ63955918 has analgesic effects on inflammatory pain tested on rats made tolerant to morphine and after peri-sciatic administration on Hargreaves thermal pain. This case illustrates that a high selectivity (more than 100x) and potent inhibition of Nav1.7 over other Nav isoforms seems to be required for safety treatment of pain.

Several other peptides are potent inhibitors of Nav1.7 but they also block other pain-related channels. A peptide isolated from *Davus fasciatus*, Df1a (μ-TRTX-Df1a, 34 amino acids), with analgesic properties on acute pain, potently inhibits several hNav subtypes (IC_50_ 2-14 nM) and all hCav3 isoforms with more potent effects on hCav3.1 and hCav3.3 [241].

β-TRTX-Cd1a (33 amino acids) extracted from the African rear-horned baboon tarantula *Ceratogyrus darlingi* (IC_50_ 16 nM on hNav1.7 and 3µM on Cav2.2), produces antinociceptive effects (0.1-10 µM; 40 µL *ipl*) on acute pain induced by the scorpion peptide OD1 [242]. Two peptides, Tap1a and Tap2a, recently purified from the venom of the Venezuelan Pinkfoot Goliath tarantula, *Theraphosa apophysis* block pain related Nav and Cav channels. Intracolonic administration of Tp1a, the most potent inhibitory peptide, almost completely reduced mechanical hyperalgesia in a model of chronic visceral pain in mice [243].

The peptide μ-TRTX-Hl1a, purified from *Cyriopagopus lividus* (former *Haplopelma lividum*) venom, is one of the only selective peptides for the TTX-R Nav1.8 channel. Hl1a, which does not belong to any NaSpTx family, inhibits Nav1.8 with a rather low affinity (IC_50_ 2 µM) but shows antinociceptive effects in inflammatory, visceral and neuropathic pain models in mice [244]. Others less selective peptides such as CcoTx1 (β-TRTX-Cm1a) and CcoTx2 (β-TRTX-Cm1b), isolated from *Ceratogyrus marshalli* (former *C. cornuatus*), are known to block Nav1.8 or Nav1.1 with similar affinities [245].

*Inhibition of Cav channels*

Spiders have developed essentially paralyzing venoms to capture their prey, mainly invertebrates, with toxins that block the entry of calcium into cells by binding to Cav channels. These toxins contribute to inhibit the release of neurotransmitters at the neuromuscular junctions leading to flaccid paralysis in the insect. Some spider toxins are also effective on mammalian Cav including those, such as Cav2.2, Cav2.3 and Cav3.2 that are involved in the transmission of pain. 

Several peptides from *Phoneutria nigriventer* venom have been isolated from the third chromatographic fraction (PhTx3). Three sub-fractions inhibit neuronal Cav and induce analgesia in different pain models in rodents [36,165].

Pn2a (ω-CNTX-Pn2a or PnTx3-3, 76 amino acids) one of the most toxic peptide, produces a flaccid paralysis after injection into mice [246]. Pn2a inhibits high voltage-activated Cav with the following order of efficacy Cav2.1>Cav2.3>Cav1.4>Cav2.2. It has a potent affinity for Cav2.3 and Cav2.1 (IC_50_ 12-16 nM) but also irreversibly blocks (Kd =0.7 nM) Cav2.2 channels [247-249]. Central supraspinal (*icv*) and spinal (*it*) injections of Pn2a (30 pmol) in rodents induce short analgesic effects in acute thermal (tail flick) pain models without motor dysfunction [250,251]. Spinal injection of Pn2a also induces long-lasting antinociceptive effects in mechanical allodynia produced in neuropathic pain models without impairing functions at higher doses. However, the same injection of Pn2a does not alter mechanical sensitivity in non-neuropathic pain models. Moreover, spinal (*it*) injection of Pn2a does not prevent or reverse mechanical allodynia in inflammatory models, revealing that Cav2.1 and Cav2.3 channels are probably not involved in this inflammatory pain whereas they are involved in neuropathic pain. In addition, the inhibition of glutamate release linked to Cav2.1 and Cav2.3 blockade could be an explanation for the antinociceptive effect of Pn2a by both supraspinal and spinal injections [250,251]. 

In the same way, Pn3a (ω-CNTX-Pn3a or Tx3-4), another peptide isolated from *Phoneutria* venom, and known to block Cav2 channels, inhibits capsaicin-stimulated release of glutamate in Ca^2+^ dependent and independent ways [252]. Spinal injection of Pn3a (30 pmol/site, *it*) seems to have no effect on acute nociceptive pain in hotplate test. However, Pn3a (until 100 pmol/site, pre- or post-administered *it*) reversed nociception of the second inflammatory phase in a formalin pain model in mice without motor side effects [253]. Spinal Pn3a injection (3 pmol/site) also improves pain recovery in a post-operative model tested by mechanical hypersensitivity.

The third Cav blocker purified from *Phoneutria nigriventer* venom is Pn4a (ω-CNTX-Pn4a also called Phα1β or PnTx3-6) (55 amino acids) the most abundant peptide. Pn4a blocks Cav1.2, Cav2.1, Cav2.2 and Cav2.3 channels with a relative selectivity for Cav2.2 (IC_50_ 136 nM) [254]. Pn4a (*it* injections in rats) reduces pain behaviors in inflammatory pain models (formalin and CFA tests), where the contribution of glial cells in the dorsal horn of spinal cord to allodynia has been shown [255]. During inflammation, Pn4a also have analgesic effects in irritating pain induced by capsaicin [256-258] where it reduces spontaneous and mechanical allodynia [259]. Pn4a induces a long lasting analgesia in postoperative pain with a reduction of mechanical allodynia [260,261]. In neuropathic pain (ligation of the sciatic nerve and drug-induced fibromyalgia), Pn4a (200-300 pmol, *it*) reduces the mechanical hyperalgesia [256,258,262-264]. Pn4a (100 pmol/site) has also analgesic effects in visceral pain (*ip* injections of acetic acid, intracolonic injection of capsaicin) [259]. In these studies, the analgesic effect of Pn4a was compared with that of ω-conotoxin MVIIA (ziconotide, PRIALT®), a cone snail peptide known to block Cav2.2, and used as a medication in clinics to treat severe chronic pain in humans [265]. Ziconotide is however known to have narrow therapeutic windows and to induce serious side effects [266]. Pn4a has a wider therapeutic index than ω-conotoxin MVIIA, making this peptide an interesting therapeutic approach due also to its long duration of action and the absence of toxic side effects [263,267]. 

The ω-agatoxin-IVA (ω-Aga-IVA or ω-AGTX-Aa4a; 48 amino acid) is a selective and high affinity Cav2.1 blocker extracted from the venom of *Agenelopsis aperta*. ω-Aga-IVA acts in a voltage-dependent manner, and bind to a region near the voltage sensor domain IV [268]. The peptide was tested in a model of inflammation induced by injection of kaolin and carrageenan into rat knee joints where sensory neuron responses were recorded. The application of ω-Aga-IVA (0.1 µM) on the spinal cord prevented the responses to innocuous and noxious pressure onto the knee. This suggests that P-type calcium channels (Cav2.1) are involved in the generation of inflammation-evoked hyperexcitability of spinal cord neurons [269,270]. In a test measuring heat hyperalgesia on the same model of inflammation, application of ω-Aga-IVA before the induction of inflammation is only effective on secondary heat hyperalgesia [271]. This supports the thesis that spinal Cav2.1 channels are engaged in responses to noxious stimuli once, and only if, the central sensitization is established.

Group II ω-agatoxins are blockers of other pain-related Cav, *i.e*. Cav2.2 for ω-Aga-IIA and ω-Aga-IIB, whereas group III ω-agatoxins (ω-Aga-IIIA, -IIIB, -IIIC and -IIID) are less specific since they block Cav2.2 and Cav2.1 [272]. The ω-Aga-IIIA, a 76 amino acid peptide has potent effects on Cav2.1 and Cav2.2 but also on neuronal and cardiac Cav with nanomolar affinity [272,273]. Inhibition of Cav2.1 and Cav2.2 currents by ω-Aga-IIIA is partial (70%), revealing a voltage-dependence mechanism and effects are slowly reversible [274]. These peptides that are not specific for pain-related Cav have not been investigated in *in vivo* pain experiments. More specific and shorter peptides isolated from *Conus* species, such as ω-conotoxins (-GVIA, -MVIIA, -MVIIB) are extensively used to characterize the role of Cav in pain modalities.

A selective inhibitor for Cav2.3, SNX482, was isolated from the venom of the tarantula *Hysterocrates gigas*. SNX482 (41 amino acid) blocks Cav2.3 channels (IC_50_ 30nM), the so-called “R-type” current in rat central neurons, without effect on other Cav [275]. The antinociceptive effect of SNX482 was demonstrated in a chronic neuropathic SNL model in rats showing the contribution of Cav2.3 in neuropathic pain [69]. Recent work has shown that SNX482 is not so specific among all ion channels since it potently blocks the potassium Kv4.3 channels (IC_50_ <3nM) [276]. Other spider toxins, like ω-grammotoxin SIA targeting Cav2.1 and Cav2.2, or hanatoxin that blocks Kv2, have promiscuous effects because they share structural sequence homologies and they bind to a highly conserved voltage sensing domain on Cav and Kv channels [202]. 

The tarantula venom, *Cyriopagopus schmidti* (former *Haplopelma or Ornithoctonus huwena*) provided a rich source of toxins active on pain-related calcium channels. HWTX-I (μ/ω*-TRTX-Hh1a*), the major 33 amino acid peptide, is a selective inhibitor of Cav2.2 (IC_50_ 55-100nM) [277]. In inflammatory pain models (formalin test and rheumatoid arthritis in rats), central injections of HWTX-I (0.1 to 0.5 µg/kg, *it*) induces analgesia without other side effects [278,279]. Another shorter peptide, HWTX-X (28 amino acids), sharing some sequence homologies with ω-conotoxins, blocks Cav2.2 channels (IC_50_ 40 nM) [280]. However, the effects of HWTX-X have not yet been tested on pain models. A third peptide, ω-huwentoxin-XVI (HWTX-XVI or ω-TRTX-Cs16a; 39 amino acids), specifically blocks Cav2.2 (IC_50_ 60nM) and induces antinociception in several pain models in rodents. Peripheral injections of ω-HWTX-XVI (56-112 nmol/kg, *ip*) reduce heat pain (hot plate model) and inflammatory pain (formalin-test) in mice while intramuscular injections reduce mechanical allodynia in a post-operative model in rats [281]. The peptide specificity was checked on TTX-S and TTX-R Nav channels in rat DRG, and on several Kv channels that are all insensitive to HWTX-XVI. 

*Inhibition of ASICs*

Evidences on the physiological and physiopathological roles of ASIC channels were obtained by combining genetic studies with pharmacological approaches. Small molecules that are poorly specific ASIC blockers, like amiloride, benzamil or A-317567, induce analgesic effects after *in vivo* administration in rodent pain models and in some rare small clinical studies in humans [282]. But more specific ASIC antagonists like animal venom peptides allowed to demonstrate the role of ASICs in different models of acute, inflammatory, post-operative and neuropathic pain [73,77,78,283]. 

The first pharmacological tool, PcTx1 (π-TRTX-Pc1a, 40 amino acids), is a minor component isolated from *Psalmopoeus cambridgei* venom, and shares no more than 28% sequence identities with other known spider toxins that target Nav, Cav or Kv channels [284]. PcTx1 blocks ASIC1a-containing channels with a potent affinity and selectivity [283]. At physiological pH, PcTx1 is able to be an agonist of rASIC1b and of chicken ASIC1a [285,286]. PcTx1 is a state-dependent modulator, involving the pH-dependent properties of ASIC channels but also the pH at which the toxin is applied [287]. PcTx1 binds into the acidic pocked (the pH-sensor) of ASIC1a and induces a stabilization of the inactivated state of the channel by shifting its inactivation curve towards alkaline pH [286,287]. The regulation of inactivation of ASIC1a by PcTx1 occurs through the Palm and β-ball domains of ASIC1 channel, which are part of the large extracellular loop [288]. Central (*it* and *icv*) injections of PcTx1 induce potent analgesic effects in acute pain as well as in inflammatory and neuropathic pain models [76] through blockade of ASIC1a homomeric channels, and probably also ASIC1a2b heteromers [79]. These analgesic effects involve the activation of the endogenous enkephalin pathway. PcTx1 (*it*) also prevents chronic abdominal pain in a rat model of irritable bowel syndrome induced by butyrate [289].

A shorter peptide, Hm3a (π-TRTX-Hm1a), isolated from the tarantula *Heteroscodra maculata* presents high sequence identities (82%) with Pctx1 and similar effects, i.e., a potent pH-dependent inhibition of ASIC1a and potentiation of ASIC1b [290]. Hm3a has the advantage of very high biological stability that will allow the study and development of interesting tools targeting ASICs for the study of pain. 

The third spider toxin active on ASIC channels is an atypical one, Hi1a (π-TRTX-Hi1a), composed of two peptides with a short linker, presenting ICK structure and sequence identities (50 and 62% for each peptide) with PcTx1. Hi1a partially inhibits rASIC1a and hASIC1a with high affinity in a pH-independent manner and slow off-rate without effects on ASIC1b [291]. Its mode of action is different from that of PcTx1, since it binds to and stabilizes the closed state of ASIC1a. Hi1a was not tested *in vivo* on pain but in a model of stroke induction in rats, where it strongly attenuates brain damage. Hi1a could be considered as a lead for development of neuroprotective agents against brain ischemic injury.

*Interaction with TRP channels*

While TRP channels are widely distributed at peripheral nerve endings and in particular on nociceptive fibers, a few animal toxins modulate them. 

The acylpolyamines AG489 and AG505, isolated from *Agelenopsis aperta* venom, were the first animal toxins described to block TRPV1 [292]. AG489 and AG505 (1-10µM) dose-dependently inhibit TRPV1 current activated by capsaicin in a voltage-dependent manner and through a pore-blocking mechanism. Their putative analgesic effects on thermal local pain in rodents have not yet been investigated.

The 34 amino acids peptide GsMTx-4 (M-TRTX-Gr1a) isolated from *Grammostola spatulata* (*G. rosea*) blocks TRPC6, a sensor of mechanically and osmotically induced membrane stretch, which is predominantly expressed in smooth muscles but also in other cells [293]. A functional interaction between TRPC6 and TRPC1 with TRPV4, which are coexpressed in DRG neurons, contributes to the mechanism mediating primary afferent nociceptor sensitization and mechanical hyperalgesia [294]. GsMTx4 likely acts by inserting in the outer leaflet of the membrane and modifying the channel boundary lipids to favor the closed state [293,295]. Conversely, other TRP channels like TRPA1 can be activated by the toxin at 1µM according to a mechanism similar to trinitrophenol, a membrane crenator [296]. GsMTx-4 is also a modulator of stretch-activated channels (SACs), the molecular sensors for mechanotransduction, i.e., for touch, pressure, proprioception, and pain [295]. Intradermal injection of GsMTx-4 (up to 1µg) in rat hind paw, reverses hyperalgesia to mechanical and hypotonic stimuli in inflammatory and neuropathic pain models [294,297]. Mechanical pain, often caused by surgery, burns, inflammation, neuropathies, requires specific treatments not available yet in clinic, and GsMTx4, via peripheral administration, offers a new hope to alleviate this type of pain in patients.

The *Phoneutria nigriventer* venom peptide Pn4a (Phα1β) known to inhibit pain-related Cav channels is also a blocker of TRPA1 with a large difference in affinity for the rat and human isoforms [264]. In this study, authors report that central (*it*) and peripheral (*ip*) injections of low doses of the Pn4a (< 300 pmol) reduce acute nociception, mechanical and cold hyperalgesia induced by allyl isothiocyanate and the neuropathic pain evoked by the chemotherapeutic drug bortezomib.

Another *Phoneutria* peptide, Tx3-5 (U7-CNTX-Pn1a) was recently characterized as a specific and potent inhibitor of TRPV1 channels without effect on TRPA1 [298]. Tx3-5 presents interesting analgesic properties in various mouse pain models. First, local injection of Tx3-5 (100 fmol, *id*) prevents the nociceptive behavior induced by capsaicin injection into a rat left vibrissa in an orofacial test [298]. This local antinociceptive effect can be easily attributed to its blocking effect on TRPV1. Other analgesic effects are described after central injection (3-300 fmol, *it*) of Tx3-5 to mice that can prevent or reverse post-operative nociception in a dose-dependent manner [299]. Low doses (30 fmol) of Tx3-5 also induces a partial analgesia in neuropathic pain model but also in a cancer model of nociception, as potently as morphine and without adverse effects [299]. 

The ProTx-I peptide that block pain-related Nav channels also inhibits TRPA1 currents with an affinity of 390 nM without affecting TRPV1. Its high affinity for Nav1.2 was used to conduct structure-function studies with chimeric channels and ProTx-I variants. It reveals that ProTx-I inhibits both Nav and TRPA1 by binding to their S1-S4 gating domains and stabilization of the closed conformation [300]. Interestingly, mutagenesis yielded two ProTx-I variants that acquire selectivity and are only active either on TRPA1 (variant W5A) or on Nav1.2 (variant S22A) [300].

*Spider toxins and other pain-related channels*

Purotoxin-1 (PT1) (35 amino acids, ICK structure) isolated from a *Geolycosa* sp. spider, potently (IC_50_ 12 nM) and selectively blocks P2X3 ionotropic receptors in their desensitized state in rat DRG neurons. PT1 is active on homomeric forms of P2X3, and has no effect on other ion channels like TRPV1. When PT1 is injected (0.5 nmol, *ipl*) in rat hind paw, it reduces the thermal hyperalgesia triggered by inflammatory drugs such as CFA and carrageenan. It also reduces the nociceptive behavior induced by formalin in the second phase and after capsaicin injection [301]. 

The peptide GsMTx4, already known for its effects on TRP channels, blocks Piezo1 channels in its closed state, with a binding affinity of 155 nM [302]. It also inhibits human Piezo2 response to mechanical force in transfected cells and the native Piezo-like current recorded in enterochromaffin cells in gastrointestinal epithelium [303,304]. GsMTx4 acts as a gating modifier inducing a rightward shift in the pressure-gating curve, and inserts in lipids that surround the channel to favor its closed conformation [302]. The ability for many spider gating-modifiers toxins to partition into membranes appears to be an essential feature for their pharmacological activity. GsMTx-4 has the ICK motif and is amphipathic, with a hydrophobic surface, surrounded by charged residues, able to bind and penetrate the lipid bilayer, being a key to its mechanism of action on ion channels. GsMTx4 antinociceptive effects, described during inflammation-induced mechanical hyperalgesia, could be linked to its activity on Piezo channels [297].

Glutamate ionotropic receptors are permeable to cations and blocked by various polyamines isolated from spiders [305]. The AMPA type, localized on GABA neurons in spinal cord, are blocked by JSTX, a polyamine toxin isolated from the Joro spider *Nephila clavata* [306]. In a model of secondary hyperalgesia generated by a first-degree burn on rat heel, central administration of JSTX (5µg, *it*), before the burn, blocks mechanical allodynia measured on the plantar surface of the paw. JSTX also blocks induction of thermal hyperalgesia and mechanical allodynia on an inflammatory model induced by carrageenan, but has no effect in the formalin test [307]. These data show the important role of AMPA receptors in the regulation of hyperalgesia induced by tissue injury and inflammation.

The toxin PnTx4(5-5) (Γ-ctenitoxin-Pn1a, 47 amino acids) isolated from *Phoneutria nigriventer* venom has a high insecticidal activity but no toxicity after *icv* injections in mice. PnTx4(5-5) (100 nM) blocks NMDA evoked currents, reducing EPSCs in rat hippocampal slices, thus showing neuroprotective effects against glutamate-mediated excitotoxic neuronal cell death [308]. The peptide does not affect GABA, kainic acid or AMPA receptors. In a more recent study [309], local injections of PnTx4(5-5) (5µg, *sc*) were shown to reduce hyperalgesia induced by PGE2 or carrageenan in rats, in a dose-dependent manner. PnTx4(5-5) also reverses glutamate-induced hyperalgesic effects, showing a clear relation between analgesia and its effects on glutamatergic system [309].

## Interaction of Toxins with Phospholipids

Experimental evidence suggests that voltage-gated ion channels are located in the cell membrane within raft domains, regions that are very rich in cholesterol and sphingomyelin and display unique physical properties. This location is considered to be important for the pharmacological sensitivity of ion channels for toxins. More and more toxins are identified in which the interaction with lipids promotes the binding, selectivity and affinity for ion channels.

Several spider peptides acting as gating modifiers have an amphipathic nature and present an ability to bind to lipid membranes, correlated to their affinity for the targeted ion channel [310]. Studies have proposed that they form a tri-molecular interaction with lipids and voltage-gated ion channels. Spider toxins active surface is generally composed of a conserved hydrophobic patch surrounded by a charged ring of amino acids. This active surface promotes not only the interaction with ion channels but also with membrane lipids via hydrophobic interactions. The charged ring generates electrostatic interactions with the phospholipid headgroups. In a study set out to determine whether lipids interact with the voltage-sensing S1-S4 domains in Kv channels, it was shown that sphingomyelin interacts within a particular motif within voltage sensors [311]. In particular, the S3b-S4 paddle motif determines the sensitivity of the channel to lipid modification. Thus, both lipids and spider toxins interact with the paddle motif, thus defining a triangular interaction. As evidenced, the lipid modifications, by exposure to sphingomyelinase D, alters voltage sensor pharmacology to spider toxin and mutations in the paddle motif alters toxin affinity.

Spider ICK peptides like GsMTx4, or the Kv blockers SGTx1 and HpTx2 that act as gating modifiers interact with different mode on lipid bilayer [312,313]. GsMTx4, which is amphipathic, makes hydrophobic and electrostatic interactions with lipids. GsMTx4 alters bilayer mechanical properties so that it may disturb the lipid packing adjacent to the channel. Thus, GsMTx4 can selectively inhibit the gating of cation selective SACs by increasing the membrane tension required for activation [314].

Other examples are given with spider ICK toxins acting on pain-related-Nav channels, such as ProTx-II, which has a potent affinity for hNav1.7 (IC_50_ 0.3 nM) and good lipid membranes-binding properties [315]. It has been shown that there is a direct correlation between ProTx-II membrane binding affinity (to the water-lipid interface) and its potency as a hNav1.7 channel inhibitor. ProTx-II analogues, with substitution of hydrophobic aromatic Trp residues by nonaromatic amino acid residues, possess a lower tendency to bind/insert into the membrane, thus showing reduced binding affinity for lipid membranes in relation to a decreased inhibition potency for hNav1.7. This indicates that each of the Trp and Lys residues are important for the membrane-binding properties of ProTx-II. In addition, an increase in overall positive charge enhances the membrane binding affinity of ProTx-II for membranes, probably due to increased electrostatic attractions for the phosphate groups in the phospholipid headgroups [315].

In contrast to ProTx-II, HWTX-IV lacked the ability to partition into phospholipid bilayer of artificial membranes. However, the synthesis of a HWTX-IV analogue (gHwTx-IV), with only 4 amino acid substitutions in N- and C-terminal positions, increased its ability to bind to lipid membranes and also improved inhibitory potency at hNav1.7 [316,317]. The mutant gHwTx-IV has more positive charges on the face possessing the four mutations that allows electrostatic interactions with the membrane. The mutant also has a notable increase in the hydrophobic area due to the presence of Gly and Trp substitutions allowing additional hydrogen bonding and hydrophobic interactions of gHwTx-IV, compared to the native HwTx-IV, with hNav1.7.

The 33 amino acid peptide μ-TRTX-Hhn2b (HNTX-I), isolated from *Cyriopagopus* (former *Haemadipsa* or *Ornithoctonus*) *hainanus* venom is inactive on TTX-S and TTX-R mammalian Nav, but the production of a double mutant of this peptide (G7W; N24S), which creates subtle displacements of the side chains of key pharmacophore residues, allows a potent inhibition of hNav1.7. In particular, one of these substitutions (G7W) creates a selective binding to anionic lipids [318]. Other substitutions that reduce negative charges, and enhance positive charges surface but also strengthened hydrophobic interactions, result in a clear increase of activity on Nav1.7 channels [319]. Some mutation studies show that critical residues represented by the motif X1X2SWCKX3 are required for the activity on Nav1.7, X representing a hydrophobic residue and S required to position W and K correctly on the active surface of spider peptides [320,321].

## Toxins as a Basis for Developing New Pain Treatments

Pain is one of the first reasons for consulting a general practitioner. Acute pain is a protective signal while chronic pain, if it lasts more than 3 months, becomes a real pathology. This is the case of neuropathic pain resulting from internal nerve damage in cases of trauma or during inflammatory, infectious, or metabolic diseases [322]. It also occurs during cancer treatment with chemotherapy. Whatever its duration, intensity and etiology, pain deserves an adapted treatment with tolerance and the minimum of undesirable side effects [323].

Among the treatments currently used, acetaminophen, nonsteroidal anti-inflammatory drugs, serotonin-noradrenaline reuptake inhibitors, tricyclic antidepressants, antiepileptics are often used as first-line drugs. Weak opioids are prescribed as second line, then strong opioids as last line drugs [324]. These analgesics have found application for various types of pain but all have uncomfortable side effects in patients, related to their impact up peripheral and central nervous systems [325]. During treatment of neuropathic pain, many patients remain refractory to, or intolerant of the existing pharmacology, and some drugs also have narrow therapeutic window [326,327]. New families of drugs as well as new targets involved in pain could provide therapeutic leads for the synthesis of antinociceptive drugs. 

Cav2.2 channels that are expressed in peripheral neurons have been validated as pain target with, in particular, the feedback from the use of ziconotide in clinical trials [265]. Ziconotide is the only drug, based on animal toxin, used in clinics to treat severe intractable pain. Ziconotide has analgesic properties as potent as morphine and needs to be administered at the spinal level in patients. However, due to its narrow therapeutic index, it can cause undesirable side effects such as motor disturbances, dizziness, somnolence, amnesia, hallucinations, and nausea, but is neither addictive nor induces tolerance, unlike morphine [266]. The numerous *Phoneutria* and *Cyriopagopus* ω-toxins targeting Cav2.2 could also be used as a basis for the development of new pain medications.

In recent years, Nav1.7 has been the focus of interest and validated as a pain target based on observations of human genetic loss of function, showing inability to sense pain and only minor sensory impairment in individuals with this channelopathy [50]. Its preferential expression in peripheral sensory and sympathetic neurons makes it an ideal target for analgesics [193]. 

Spider venoms are rich in modulators for pain targets, so it is not surprising that recent research has aimed to modify and develop new toxin structures to improve the specificity and affinity of their interactions with ion channels of interest. Spider toxins that inhibit Nav channels involved in pain also offer the advantage of being shorter than those of scorpion venoms that have the same targets. Although scorpion toxins, such as those from *Buthus martensii*, have real potential to overcome pain, particularly for cancer treatments [328,329], spider toxins are also much more specific for subtypes of pain-related ion channels (e.g. Protx, HwTx-IV, GsAF, GpTx-I, Pn3a, PcTx1...) and some have potent *in vitro* and *in vivo* effects. Alanine scanning of several spider peptides and their structure -function studies on ion channels revealed that some residues are critical for potent activity, and that substitution of other residues confers more potency and ion channel selectivity [216]. HwTx-IV is a potent blocker of Nav1.7, which also inhibits neuronal Nav1.1, 1.2, 1.3, and 1.6 channels but with a good selectivity against cardiac Nav1.5 channel. A triple mutant of HwTx-IV (m3-HwTx-IV), showed an increased potency for Nav1.7, without major structural modifications, widening the gap with its effects on the muscle Nav1.4 and no effect on cardiac Nav1.5 channels [330]. The mutant peptide also provides analgesia in a mouse model of acute pain confirming the same *in vivo* activity as the wild type peptide. Such mutations were done on several spider toxins allowing more selective and potent Nav1.7 antagonists [331].

*In vivo* stability of peptide toxins can be impaired by enzymatic degradation that make them unsuitable for oral therapeutic administration and requires intrathecal injections. Their disulfide bonds are susceptible to reduction by isomerases, leading to chain unfolding and oxidative refolding with consequent loss of biological activity. Replacement of disulfide bonds results in analogues with comparable potency. This type of modification was done with ProTx-II, where replacement of cysteine by lanthionine results in a thioether side-chain bridge that cannot be cleaved *in vivo* [332]. However, this approach was not successful in delivering potent or selective Nav1.7 channel blockers based on ProTx-II.

The compact, folded structure of disulfide rich peptides often protects them from proteolysis, but they still present risks to have a short *in vivo* half-life due to a rapid renal filtration. To avoid too rapid elimination, coupling with large proteins increasing their half-life and improving their bio-availability *in vivo* has been considered. Some examples of these approaches have been described with modified natural toxins from spiders. PEGylation, consists to add a hydrophilic polymer to the peptide of interest, without affecting its potency on ion channels. PEGylation on several residues of GpTx1, whose position is not crucial for its function on Nav1.7, showed the same potency and selectivity as compared to the native GpTx1 [333]. Another approach using multivalent peptides has been shown, in some cases, to have increased binding affinity for their targets. The explanation could be that a dimeric peptide can simultaneously bind to different sites on the target or that each peptide bridges two different channels in close proximity in the cell membrane. Improvement of bio-availability has been achieved using GpTx1 as a homodimer constituted by two peptides linked by a hydrophilic polymer (long chain PEG) that also improved the affinity and binding properties to Nav1.7 [333]. Another double knotting peptide was produced, using a chemoenzymatic ligation with sortase A, creating an amino acid link between two spider toxins [334]. CcoTx-I, CcoTx-II, HxTx-IV and their analogues known to inhibit Nav1.7 were used either in homo or hetero dimers to compare their activity and binding on Nav1.7. All dimers were active, some peptide combinations resulted in an equivalent potency compared to the individually peptide application, others resulted in intermediate potency and a more cooperative binding. Some others showed that peptides need a longer incubation time with Nav1.7 to achieve binding equilibrium [334]. The benefits of modulating Nav channels with bivalent knottins could be that peptides would stay longer at the channel binding site, leading to a sustained inhibitory effect. This would also allow an ability to modulate inhibitory potency with varied PEG linkers. Combining a ligand that binds to a voltage-sensor domain with one that binds to the pore domain has also been tried using a conotoxin (for pore occlusion) and a spider peptide, which allosterically modulates the Nav1.4 channel gating [335]. This generated a heterobivalent ligand with a higher potency and with a slower dissociation rate.

The design of cyclic peptide inhibitors is another approach for the development of promising therapeutic drugs. Using a common pharmacophore deduced from a conotoxin and a spider peptide active on two pain-related Nav1.7 and Nav1.9 channels, a short ten residue cyclic peptide was designed. Cyclization was obtained via a disulfide bond. The cyclic mutant presented not only increased inhibitory activity on Nav1.7 and Nav1.9 channels, but it also has a better serum stability [336].

Peptide-antibody conjugates are also used and confer improvements, due to the large size and hydrodynamic properties of the antibody. Their interaction with specific receptors (FcRn) decreases renal filtration and recycling. An analogue of JzTx-V, potent inhibitor of Nav1.7, was designed in this intention resulting in a JzTx-V-antibody conjugate with 100-fold improved *in vitro* potency but a reduced half-time *in vivo*. The reduction of net positive charges on the peptide helps to improve plasma exposure in rodents, ultimately resulting in a compound with moderate activity on Nav1.7 [337]. 

Another example was found using GpTxI that also inhibits Nav1.7. GpTxI analogues with amino acid substitutions improving selectivity against cardiac and muscle Nav channels, were developed, and linked using a PEG linker on a carrier monoclonal antibody [338]. These modifications confer an extended half-life but only moderate *in vitro* activity and exhibited no activity in a mouse histamine-induced pruritis model. The potency of the peptide−antibody conjugates was dependent on the conjugation site within the antibody, the length of the linker, and the peptide loading.

## Conclusion

Scorpion and spider venoms offer a huge amount of toxins that can explain their neurotoxicity and pain-inducing effects. Toxins able to modulate specific ion channels implicated in pain are certainly important actors for pain perception and integration in mammals during envenomation, even if other components - i.e. small molecules like histamine, serotonin, or ATP -also contribute to nociception.

In recent years, an increasing number of toxins have been discovered that modulate pain-related channels, which help us to better understand their biophysical properties and roles. However, in the search for new analgesics careful attention must be given, since the same venom peptide can target several channels in the same family, thus inducing *in vivo* undesirable effects. The same toxin can act differently on ion channels of different animal species (i.e. insects or vertebrates), which precludes foreseeing effects in humans. The case of δ-hexatoxins that are toxic to humans and primates but not to other vertebrates (cats and dogs) is an example. The same toxin can also act in opposite mechanisms in the same family of ion channels, as illustrated by HpTx1 on Nav1.7 and Nav1.9 channels. With the same venom, several toxins can also act on different ion channels in the same way, that means synergizing their *in vivo* effects, or in an opposite manner, as it has been shown for PcTx1 and VaTx peptides isolated from *Psalmopoeus cambridgei*. Then, the question about the role of these toxins in the predation or defense for spiders remains elusive. 

The presence of a minority of peptides having opposite effects compared to other majority of venom peptides could suggest that the less abundant ones are traces of ancestral toxins that have disappeared in the course of evolution in order for the animal to adapt to its environment, its prey as well as its aggressors. The way of venom or toxin injection is also determinant for toxic effects. Usually, stings or bites in vertebrates primarily affect the peripheral nervous system and it is not well known whether long toxins are able to cross the blood-brain barrier. When spiders hunt their prey, they seek to bite the insect at the nerve centers, which they locate perfectly, for a lightning action, or elsewhere if they want to keep their prey alive. In the latter case, analgesic toxins may help the spider (or scorpion) to calm and control its prey for easier feeding.

Pain is the most frequent symptom during scorpion and spider envenomation, but curiously, various toxins with the opposite effect, rather promoting antinociceptive action, have been found in venoms. More and more peptides with analgesic properties are actually used as molecular templates with structural modifications to improve their pharmacological profile, with higher selectivity and affinity, and their *in vivo* bio-availability to propose new analgesic drugs to human medicine. Some clinical trials with selective drugs for pain-related channels are in progress with promising results.

### Abbreviations

ASIC: acid sensing ion channels; Cav: voltage-gated calcium channel; CNS: central nervous system; DRG: dorsal root ganglia; EPSC: excitatory post-synaptic currents; h: human; *icv*: intra cerebro ventricular; *id*: intradermal; *ip*: intraperitoneal; *ipl*: intraplantar; *it*: intrathecal; *iv*: intravenous; IC_50_: toxin concentration necessary to inhibit 50% of the response; ICK: inhibitory cystine knot; KO: knockout; Kv: voltage-gated potassium channel; NaSpTx: voltage-gated sodium channel spider toxin; Nav: voltage-gated sodium channel; NMDA: N-methyl-D-aspartate; PNS: peripheral nervous system; r: rat; TTX: tetrodotoxin; TTX-R: resistant to tetrodotoxin; TTX-S: sensitive to tetrodotoxin.
